# The oncogenic fusion protein TAZ::CAMTA1 promotes genomic instability and senescence through hypertranscription

**DOI:** 10.1038/s42003-023-05540-4

**Published:** 2023-11-18

**Authors:** Emily Neil, Roberto Paredes, Oscar Pooley, Brian Rubin, Valerie Kouskoff

**Affiliations:** 1 Developmental Hematopoiesis Group, Faculty of Biology, Medicine and Health, the University of Manchester, Manchester, M13 9PT UK; 2 Department of Cancer Biology, Lerner Research Institute, Cleveland Clinic Foundation, Cleveland, OH 44195 USA

**Keywords:** Cancer, Oncology

## Abstract

TAZ::CAMTA1 is a fusion protein found in over 90% of Epithelioid Hemangioendothelioma (EHE), a rare vascular sarcoma with an unpredictable disease course. To date, how TAZ::CAMTA1 initiates tumour formation remains unexplained. To study the oncogenic mechanism leading to EHE initiation, we developed a model system whereby TAZ::CAMTA1 expression is induced by doxycycline in primary endothelial cells. Using this model, we establish that upon TAZ::CAMTA1 expression endothelial cells rapidly enter a hypertranscription state, triggering considerable DNA damage. As a result, TC-expressing cells become trapped in S phase. Additionally, TAZ::CAMTA1-expressing endothelial cells have impaired homologous recombination, as shown by reduced BRCA1 and RAD51 foci formation. Consequently, the DNA damage remains unrepaired and TAZ::CAMTA1-expressing cells enter senescence. Knockout of *Cdkn2a*, the most common secondary mutation found in EHE, allows senescence bypass and uncontrolled growth. Together, this provides a mechanistic explanation for the clinical course of EHE and offers novel insight into therapeutic options.

## Abstract (web summary)

Expression of the oncoprotein TAZ::CAMTA1 leads to hypertranscription and considerable DNA damage. As TAZ::CAMTA1-expressing cells have impaired homologous recombination, the DNA damage remains unrepaired and the cells enter senescence.

## Introduction

Epithelioid Hemangioendothelioma (EHE) is a rare sarcoma of vascular endothelial cells, with an unpredictable disease course^1^. EHE is a heterogeneous disease, with tumours reported at multiple anatomic sites, however, the lungs, liver and bones are most commonly affected^2,3^. Two mutually exclusive chromosomal translocations define EHE tumours, each involving one of the transcription co-factors TAZ and YAP1^4–6^. TAZ and YAP1 are downstream effectors of the Hippo pathway, and primarily initiate transcription by interacting with TEAD transcription factors, although they also initiate transcription by interacting with other transcription factors such as RUNX, PAX, TBX and SMAD factors^7,8^. YAP and TAZ are frequently activated in cancer, however, rarely directly mutated^4^. TAZ::CAMTA1 (TC), resulting from a t(1;3) translocation, is present in 90% of EHE tumours^5,9^. The remaining 10% of EHE cases harbour a t(X;11) translocation, resulting in the YAP1::TFE3 (YT) fusion protein^6^. It is thought that TC and YT represent the initiating events in EHE, however, secondary mutations are present in at least 55% of cases and are associated with more aggressive disease^10,11^.

TC contains the N-terminus of TAZ fused to the C-terminus of CAMTA1^5^. TC retains the TEAD binding domain, most of the WW domain and the S89 residue critical to the regulation of wild-type TAZ activity. When the Hippo pathway is active, S89 is phosphorylated by LATS1/2 leading to the inactivation of TAZ^5,12^. CAMTA1 is a calmodulin-binding transcription factor, however, its DNA binding domain is not present in TC^5,13^. Initial studies have shown TC to be less sensitive to negative regulation by the Hippo pathway, despite S89 phosphorylation^5,14^. This is due to its increased nuclear localisation, driven by the nuclear localisation signal (NLS) contributed by CAMTA1^14^. Due to its interaction with TEADs, the transcriptional programme of TC bares some similarities with the canonical TAZ signature^5,11,15^. The CAMTA1 moiety contributes by recruiting the ATAC complex and increasing chromatin accessibility^16^; this results in the EHE transcriptome being distinct from other endothelial neoplasms, but being conserved between EHE mouse models and human disease^11^.

Genomic instability is a hallmark of cancer, whereby increased DNA damage allows mutations to accumulate^17^. In cancer, DNA damage can arise from multiple sources, including replication stress^18^. Hypertranscription upon oncogene activation is an increasingly recognised mechanism behind this, as replication forks collide with the transcriptional machinery and R-loops^18,19^. Replication induced double strand breaks (DSBs) result in S phase arrest whilst repair takes place^20,21^. Homologous recombination (HR) is a DSB repair pathway which dominates during S phase, and is typically error-free^22^. HR involves resection of DSB ends by nucleases to create ssDNA overhangs, on which RAD51 is loaded by the BRCA2-PALB2 complex^23^. The RAD51 nucleofilament can then invade the sister chromatid which is used as a template for accurate repair^22,24^. BRCA1 is a key HR effector, forming distinct complexes which function at multiple stages in the pathway^25^. BRCA1 binding partners include Abraxas and RAP80 for recruitment to DSBs, CtIP for end resection, and BRCA2-PALB2 for RAD51 deposition^23,26,27^. BRCA1 inactivation in cancer leads to genomic instability as HR is impaired^28^.

HR deficiency in cancer can result in oncogene induced senescence (OIS)^29^. This tumour suppressive mechanism occurs when DNA damage is unable to be repaired, causing cells to undergo permanent growth arrest^30^. Senescent cells are characterised by p16 expression, distinct morphology, and the secretion of inflammatory cytokines, termed the senescence-associated secretory phenotype (SASP)^31^. The SASP recruits macrophages to clear senescent cells, however this also contributes to an inflammatory, oncogenic microenvironment when senescent cells accumulate in a tumour^32,33^.

While transgenic mouse models have demonstrated the oncogenic potential of TC, we are still lacking contextual cellular model systems to dissect the initiating molecular events driven by TC to promote EHE tumorigenesis. Here, we describe a model for generating primary endothelial cells from mouse embryonic stem cells (mESCs) harbouring a doxycycline inducible TC expression system. Upon TC induction, endothelial cells rapidly acquire transcriptomic and phenotypic features characteristic of human EHE. Using this model system to investigate how TC promotes tumour initiation, we uncover a critical role for TC in promoting genomic instability via hypertranscription and DNA damage response interference.

## Results

### TC expression induces cell cycle arrest in primary endothelial cells

**Fig. 1:**
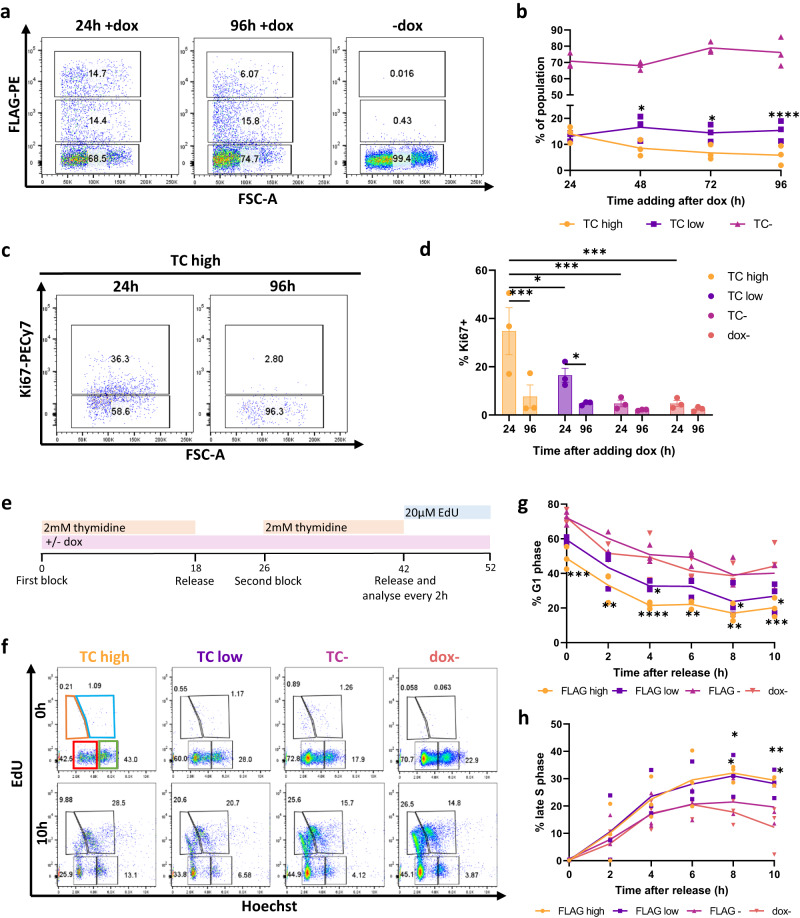
TC expression in endothelial cells initially results in cell cycle arrest. **a** Representative flow cytometry plots showing the frequency of TC expressing cells against FSC-A at 24 h, and 96 h with or without addition of doxycycline (dox) to induce TC expression. **b** The frequency of TC expressing endothelial cells at 24 h, 48 h, 72 h and 96 h after addition of dox to induce TC expression, as measured by flow cytometry, n = 4. Statistical significance was determined by two-way ANOVA with Tukey’s multiple comparisons test, n = 3. **c** Representative flow cytometry plots showing the frequency of Ki67+ endothelial cells within the TC high population, at 24 h and 96 h after dox induction. **d** The frequency of Ki67+ endothelial cells at 24 h and 96 h after dox induction, within TC high, low, negative and uninduced populations, n = 3. Statistical significance was determined by two-way ANOVA with Sidak’s multiple comparisons test. **e** Experimental timeline showing the double thymidine block protocol for cell cycle synchronisation at the G1/S phase boundary. **f** Representative flow cytometry plots showing EdU incorporation against Hoechst staining in TC high, TC low, TC- and uninduced endothelial cell populations at 0 h and 10 h after releasing cells from G1/S phase block. This reveals four populations EdU- Ho low (G1 phase; red gate), EdU+ Ho low (early S phase; orange gate), EdU+ Ho high (late S phase; blue gate), and EdU- Ho high (G2 phase; green gate). **g** Frequency of cells in G1 phase over 10 h following release from thymidine block, in TC high, TC low, TC- and uninduced endothelial cell populations, n = 3. **h** As (**g**) but for frequency of cells in late S phase, n = 3. Statistical significance was determined by two-way ANOVA with Dunnett’s multiple comparisons test. In all panels error bars show SEM, and *p < 0.05, **p < 0.01, ***p < 0.001, ****p < 0.0001.

As wild-type TAZ is known to regulate S phase entry and proliferation in endothelial cells^34^, we first determined if TC expression had a similar effect. The TIE2^+^FLK1^+^ endothelial progenitor cell population was isolated from differentiating mESCs and further cultured in endothelial promoting culture conditions (Supplementary Fig. 1a–d). The resulting cells expressed endothelial markers (TIE2, FLK1, and low level of CD31 and VE-cadherin) and formed cord-like structures in Matrigel plugs (Supplementary Fig. 1c, d). Addition of dox induced the co-expression of FLAG-tagged TC and GFP via an IRES sequence, which enables the tracking of TC-expressing cells by GFP expression in both live and fixed cells (Supplementary Fig. 1e, f). To investigate TC-expression over time, dox was added to endothelial cells and flow cytometry analysis was carried out. FLAG epitope staining revealed the presence of two distinct populations expressing low or high level of the TC protein (Fig. 1a). At 48 h post induction, there was a significantly lower frequency of TC high endothelial cells compared to TC low, despite initially being of similar proportions (Fig. 1b). The proportion of TC high cells continued to decrease throughout the time course. This was further evidenced by Ki67 expression, which marks actively dividing cells (Fig. 1c, d). TC high and low populations initially had high frequencies of Ki67^+^ cells at 24 h post dox induction compared to TC^neg^ and no dox controls. The frequency of Ki67^+^ cells was reduced significantly by 96 h within TC high and TC low populations (Fig. 1c, d, Supplementary Fig. 1g). In contrast, there was no statistically significant difference in the frequency of Ki67^+^ between 24 and 96 h in the TC negative cells and in the no dox culture. This suggests that TC-expressing endothelial cells were unable to maintain an initially high proliferation rate, leading to a reduction in the frequency of TC high cells over time.

To investigate cell cycle progression in TC expressing endothelial cells, a double thymidine block was carried out to synchronise cells at the G1/S phase boundary (Fig. 1e). Thymidine was added for 18 h to the endothelial cultures, followed by an 8-h block-release, then an additional 16-h thymidine block. At the end of the second block, cells were released and EdU was added to distinguish S phase cells. Hoechst staining was used to differentiate between G1 and G2 phase cells (Fig. 1e, f, Supplementary Fig. 2a). Flow cytometry revealed that TC-expressing populations had lower frequencies of G1 cells immediately after release compared to TC^neg^ and uninduced controls (Fig. 1g). Additionally, the proportion of TC-expressing cells in late S phase increased continually throughout the experiment, with a higher proportion than in the TC^neg^ populations by 8 h (Fig. 1h). The proportion of TC^neg^ and uninduced endothelial cells in late S phase increased steadily, then began to decline (Fig. 1h). The proportion of TC high endothelial cells in early S phase remained steady throughout the experiment, with a significantly smaller population than endothelial cells not expressing TC (Supplementary Fig. 2a, b). The proportion of cells in G2 phase decreased for all populations throughout the experiment; however, this proportion did remain significantly higher in TC high endothelial cells (Supplementary Fig. 2c). The higher proportion of TC-expressing cells in G2 phase at early time points is likely due to their decreased sensitivity to the thymidine block, another sign of cell cycle arrest. There were no significant differences for any cell cycle phase between uninduced controls and TC^neg^ endothelial populations (Fig. 1e–h, Supplementary Fig. 2).

Together, these findings suggest that while initially TC expression caused increased proliferation, endothelial cells were subsequently arrested in late S phase, resulting in a lower proliferation rate compared to TC^neg^ and uninduced cells.

### TC expression causes DNA double-strand breaks, independent of its interaction with TEAD

**Fig. 2:**
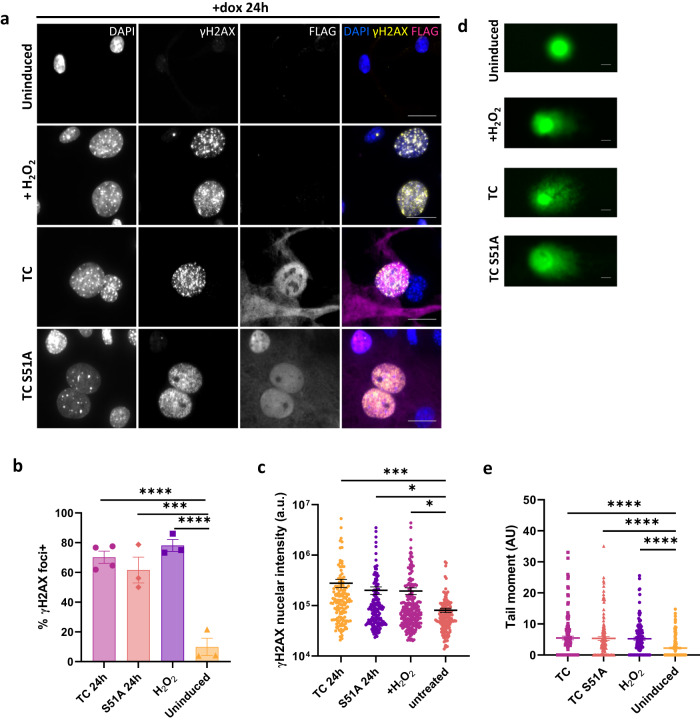
TC expression causes endothelial cells to accumulate DNA double strand breaks. **a** Representative images showing endothelial cells treated with dox for 24 h to induce TC expression or not. Cells treated with 30 μM H_2_O_2_ for 4 h were used as a positive control. Cells were stained with DAPI (nuclei; blue), FLAG antibody (TC; magenta), and γH2AX antibody (phospho-H2AX; yellow). All imaging was performed on a Zeiss fluorescence widefield microscope using a 63x oil immersion objective. Scale bars = 20 μm. **b** Frequency of endothelial cells positive for γH2AX foci from imaging experiments as presented in (**a**). Cells with more than 10 foci were considered positive. Significance was calculated by one-way ANOVA and Dunnett’s multiple comparisons test. **c** Nuclear fluorescence intensity of γH2AX staining in cells from the imaging experiments as presented in (**a**). Significance was calculated by one-way ANOVA and Dunnett’s multiple comparisons test. In imaging experiments, a minimum of 150 cells per condition were analysed, n = 4. **d** Representative images from neutral comet assay to detect double strand breaks in DNA. Day 10 endothelial cells were either left untreated, incubated with dox for 24 h to induce TC or TC S51A expression, or treated with 30 μM H_2_O_2_. Scale bars=10μm. **e** Graph showing the calculated Olive tail moment from the four conditions in (**d**). A minimum of 125 cells per condition were analysed. Statistical significance was determined by one-way ANOVA with Sidak’s multiple comparisons test, n = 3. In all panels error bars show SEM, and *p < 0.05, **p < 0.01, ***p < 0.001, ****p < 0.0001.

A well described mechanism behind S phase arrest is the accumulation of DNA damage^20,21^. Cells become arrested in S phase whilst damage is repaired to stop mutations being passed on to daughter cells. At sites of DSBs, γH2AX accumulates as H2AX is phosphorylated at S139 by the ATM/ATR kinases^21,35^. To address this potential cause for the S phase arrest observed in TC-expressing cells, endothelial cells were stained for γH2AX 24 h after induction of TC expression, to assess the present of DSBs. This revealed that TC expression resulted in a substantial increase in γH2AX positive cells, with an increased frequency of cells containing over 10 γH2AX foci and overall nuclear γH2AX signal intensity similar to that of cells treated with H_2_O_2_ (Fig. 2a, b, c). This effect was also observed upon induction of TC S51A expression, a mutant TC which cannot interact with TEAD factors^36^ (Fig. 2a, b). The lack of TC S51A interaction with TEAD was confirmed by immunoprecipitation (Supplementary Fig. 3a). γH2AX staining was already noticeable 4 h after induction (Supplementary Fig. 3b–d), suggesting that DSBs were a direct consequence of TC expression and were independent of the interaction with TEAD factors. The number of foci and overall nuclear γH2AX signal intensity in both dox and H_2_O_2_ treated populations was significantly higher than in uninduced endothelial cells at both time points (Fig. 2b, Supplementary Fig. 3).

To independently confirm the presence of DSBs in TC-expressing cells, a neutral comet assay was performed which detects DNA fragmentation, indicative of DSBs, by single-cell gel electrophoresis (Fig. 2d, e). Untreated endothelial cells were compared to cells induced with dox for 24 h or treated with H_2_O_2_ for 4 h. The tail moment was then calculated to quantify DNA damage^37^. Comet assay revealed that TC and TC S51A expressing cells, similar to H_2_O_2_ treated cells, had a significantly larger tail moment compared to untreated cells, of which most had no tail (Fig. 2d, e). These data revealed that endothelial cells acquire a large number of DSBs upon TC expression, leading to cell cycle arrest. Moreover, this effect was independent of TC interaction with TEAD factors.

### TC expression mediates hypertranscription and replication stress in endothelial cells

**Fig. 3:**
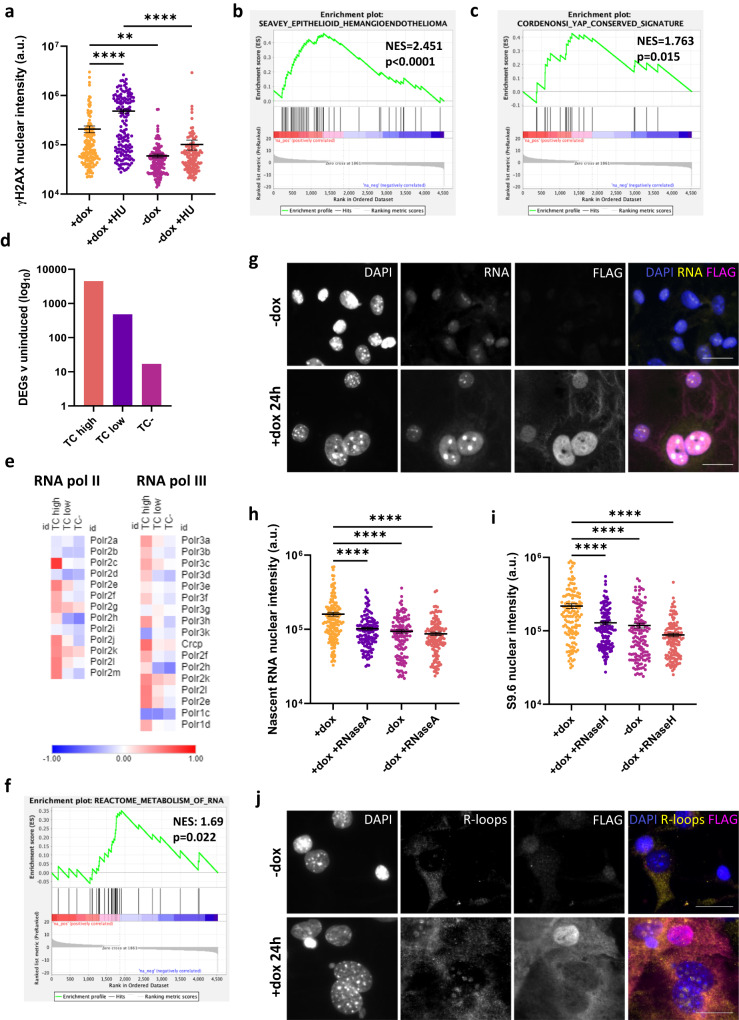
TC expression mediates hypertranscription and replication stress in endothelial cells. **a** Nuclear fluorescence intensity of γH2AX staining in dox treated or untreated endothelial samples, in the presence of hydroxyurea or not. Statistical significance was determined with a one-way ANOVA and Sidak’s multiple comparisons test, n = 3. **b** GSEA enrichment plots showing significant positive enrichment for the Seavey Epithelioid Haemangioendothelioma gene set in TC high endothelial cells. **c** GSEA enrichment plots showing significant positive enrichment for the Cordenonsi YAP conserved signature gene set in TC high endothelial cells. **d** The number of significantly differentially expressed genes (DEGs) determine from RNA-seq data in TC high, TC low and TC- populations when compared to uninduced controls. **e** Heatmaps showing log2 fold change in RNA expression of RNA polymerase II and III subunits when comparing TC high, TC low and TC- endothelial cell populations to uninduced controls. **f** GSEA enrichment plots showing significant positive enrichment for the reactome RNA metabolism gene set in TC high endothelial cells. **g** Representative images showing nascent RNA staining after endothelial cells were incubated with 5-EU for 1 h, and treated with or without dox for 24 h to induce TC expression. Cells were stained for 5-EU (nascent RNA; yellow), DAPI (nuclei; blue) and FLAG (TC; magenta). Imaging was performed on a Zeiss fluorescence widefield microscope using a 63x oil immersion objective. Scale bars = 20 μm. **h** Fluorescence intensity of nuclear 5-EU staining as shown in (**g**), to quantify nascent RNA in endothelial samples treated with dox for 24 h or not. RNaseA treatment was used as a negative control. Statistical significance was determined with a one-way ANOVA and Sidak’s multiple comparisons test, n = 3. **i** Fluorescence intensity of nuclear S9.6 antibody staining as shown in (**j**), to quantify the presence of R-loops in endothelial samples treated with dox for 24 h or not. RNaseH treatment was used as a negative control. Statistical significance was determined with a one-way ANOVA and Sidak’s multiple comparisons test, n = 3. **j** Representative images showing S9.6 antibody staining to visualise R-loops in endothelial cells treated with or without dox for 24 h to induce TC expression. Cells were stained for R-loops (yellow), DAPI (nuclei; blue) and FLAG (TC; magenta). Imaging was performed on a Zeiss fluorescence widefield microscope using a 63x oil immersion objective. Scale bars=20μm. In all imaging experiments, at least 145 cells per condition were analysed. In all panels error bars show SEM, and *p < 0.05, **p < 0.01, ***p < 0.001, ****p < 0.0001.

Next, we sought to determine the mechanism behind TC-induced DSBs. Oncogene activation can result in DNA damage via multiple mechanisms; however, we investigated replication stress due to the short time period by which DSBs occur after TC expression^18,38^. Treatment of TC-expressing endothelial cells with hydroxyurea, a known stimulus of replication stress, resulted in increased γH2AX signal compared to the populations treated with dox alone (Fig. 3a, Supplementary Fig. 4a). This suggests that replication stress is the cause of increased γH2AX expression in TC-expressing endothelial cells. Hydroxyurea had little effect on the intensity of γH2AX signal in uninduced endothelial cells (Fig. 3a, Supplementary Fig. 4a). To further investigate this, we performed RNA-seq experiments 24 h after dox addition to compare the transcriptomes of TC high, low and negative endothelial cells to uninduced controls (Supplementary Fig. 5a, b). GSEA enrichment plots established that upon TC expression, primary endothelial cells derived from differentiating mESCs acquired a transcriptomic signature characteristic of EHE tumours and had a significant enrichment for TAZ/YAP targets^11,14^ (Fig. 3b, c, Supplementary Fig. 5c, d). Transcriptomic analysis also revealed a considerable number of differentially expressed genes (DEGs) in endothelial cells 24 h after TC induction (Fig. 3d). This is suggestive of hypertranscription, a state whereby activity of RNA polymerases is increased. Hypertranscription is increasingly recognised as a source of replication stress in cancer cells, causing DSBs as a result of R-loop formation and increased replication-transcription conflicts^39,40^. Transcriptomic analysis revealed that expression of RNA polymerase II and III subunits were increased in TC expressing populations, associated with an enrichment for RNA metabolism gene set (Fig. 3e, f). Increased transcription of genes coding for proteins involved in Polymerase II complex formation to initiate transcription was also observed (Supplementary Fig. 6). Most genes coding for general transcription factors (Gtf), mediator subunits (Med) and TATA-binding proteins (TBP) or TBP-associated factors (Taf) were upregulated most noticeably in the TC high population.

To further investigate TC-induced hypertranscription, an RNA imaging assay was used to quantify nascent RNA (Fig. 3g, h). This revealed that, 24 h after induction, TC-expressing cells had a higher amount of nascent RNA in their nuclei compared to uninduced controls (Fig. 3h). Treatment of these populations with RNaseA prior to staining significantly reduced the nascent RNA signal, confirming the specificity of the staining (Supplementary Fig. 4b). Moreover, immunofluorescence microscopy also revealed increased R-loop formation in dox treated endothelial cells (Fig. 3i, j). Treatment of these populations with RNaseH prior to staining significantly reduced R-loop signal, confirming the specificity of the staining (Supplementary Fig. 4c). Similar findings for nascent RNA and R-loop increases were also observed when TC-S51A expression was induced (Supplementary Fig. 7a–d), suggesting that these initial oncogenic events are independent of TEAD interaction. Together, this provides further evidence that hypertranscription occurs upon TC expression in endothelial cells, and that replication stress is the likely mechanism behind DSB formation and cell cycle arrest.

### Homologous recombination is impaired in TC expressing endothelial cells

**Fig. 4:**
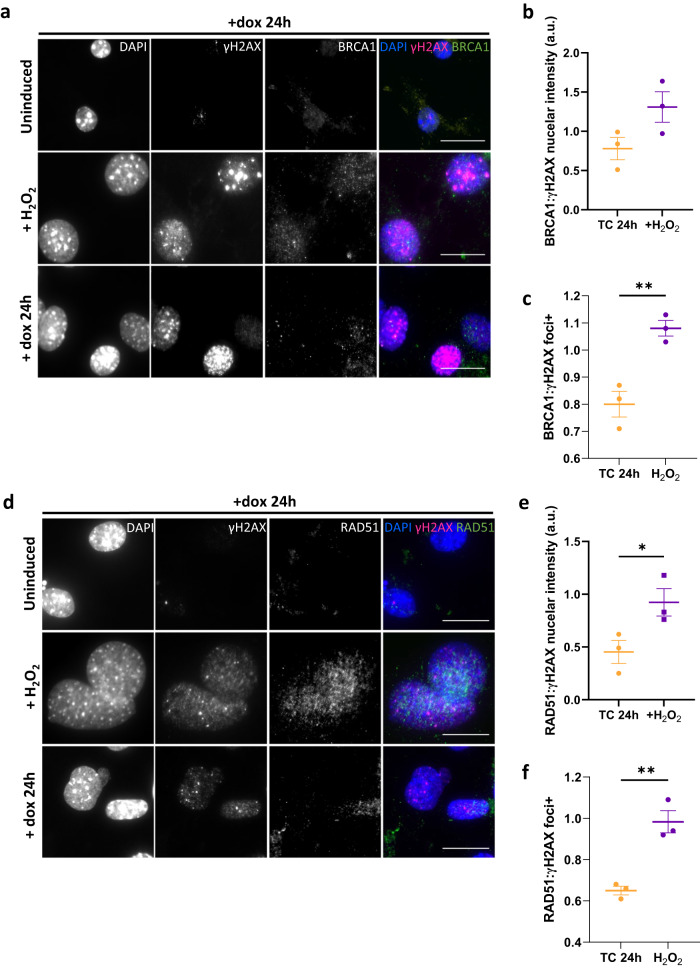
Homologous recombination is impaired in TC expressing endothelial cells. **a** Representative images showing endothelial cells that were left untreated, incubated with dox for 24 h to induce TC expression, or treated with 30 μM H_2_O_2_. Cells were stained with DAPI (nuclei; blue), γH2AX (magenta), and BRCA1 (green). **b** Ratio of mean BRCA1 to γH2AX nuclear fluorescence intensity from imaging experiments shown in (**a**). **c** Ratio of the overall percentage of γH2AX to BRCA1 foci positive endothelial cells from imaging experiments as shown in (**a**). **d** Representative images showing endothelial cells that were left untreated, incubated with dox for 24 h to induce TC expression, or treated with 30 μM H_2_O_2_. Cells were stained with DAPI (nuclei; blue), γH2AX (magenta), and RAD51 (green). **e** Ratio of mean RAD51 to γH2AX nuclear fluorescence intensity from imaging experiments shown in (**d**). **f** Ratio of the overall percentage of γH2AX to RAD51 foci positive endothelial cells from imaging experiments as presented in (**d**). All imaging was performed on a Zeiss fluorescence widefield microscope using a 63x oil immersion objective. Scale bars = 20 μm. A minimum of 150 cells per condition were quantified per experiment, n = 3. Statistical significance was calculated with an unpaired *t* test in all panels. In all panels error bars show SEM, and *p < 0.05, **p < 0.01, ***p < 0.001, ****p < 0.0001.

To understand how DSBs generated upon TC expression might contribute to EHE tumorigenesis, we next investigated the ability of these cells to repair DNA damage. As TC causes S phase arrest, and HR is the dominant pathway in S phase, we investigated the activity of key HR proteins in TC-expressing endothelial cells^22^. Using immunofluorescent imaging, we visualised the expression and localisation of BRCA1 protein, which is recruited to DSBs and mediates the formation of DNA repair complexes^25^. These experiments revealed that despite TC-expressing endothelial cells having no significant differences in BRCA1 nuclear expression (Supplementary Fig 8b), the number of BRCA1 foci^+^ per cells was reduced compared to H_2_O_2_ treated control populations, despite similar amounts of γH2AX foci (Fig. 4a–c, Supplementary Fig. 8a–d). A threshold of 10 foci/nucleus was used to define BRCA1 positivity. To further investigate this, we next determined the amount of RAD51 foci in TC expressing endothelial cells. RAD51 is downstream in the HR pathway and is recruited to the ssDNA overhangs produced after DSB end resection, where it mediates strand invasion^22,24^. RAD51 deposition is dependent on BRCA1 forming a complex with BRCA2 and PALB2^23^. This analysis revealed that in TC-expressing cells, while the nuclear RAD51 signal intensity was not significantly different to H_2_O_2_ treated control populations, γH2AX signal was significantly higher (Supplementary Fig. 8e, f). As such, the ratio of RAD51: γH2AX nuclear signal intensity was reduced (Fig. 4d, e). Again, cells with more than ten nuclear RAD51 foci were significantly less prevalent in TC-expressing cells than in H_2_O_2_ treated controls (Fig. 4d, f, Supplementary Fig. 8g, h). Both BRCA1 and RAD51 nuclear signal and foci positivity was low in uninduced endothelial cells (Fig. 4a, d, Supplementary Fig. 8). Together, these data suggest that the HR arm of DNA damage response is impaired in TC-expressing cells.

### TC expression in endothelial cells results in oncogene induced senescence and genomic instability

**Fig. 5:**
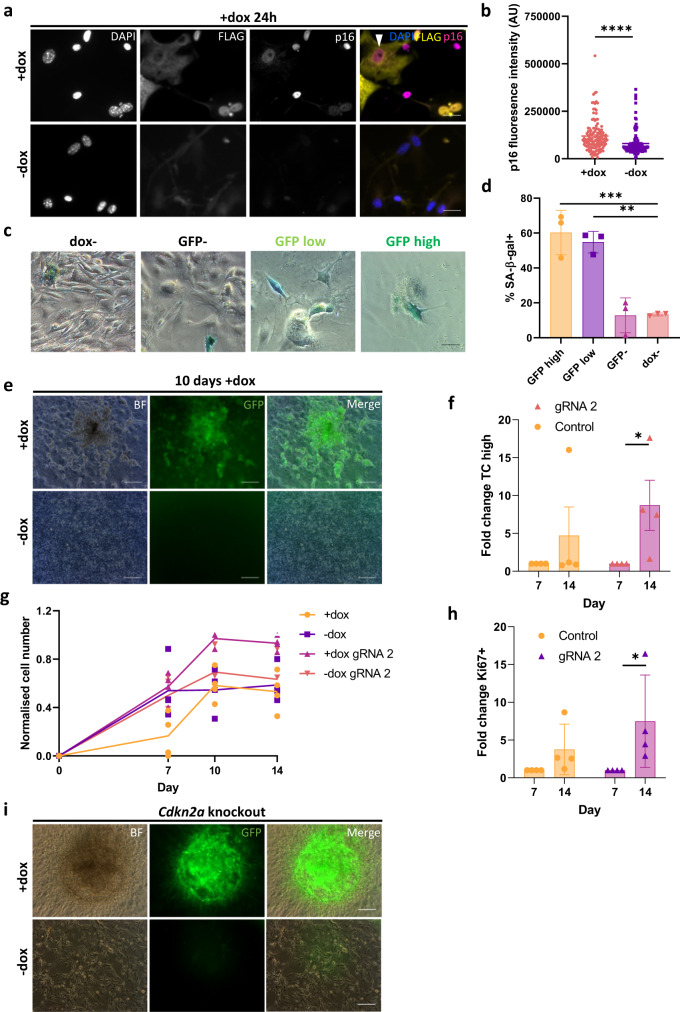
TC expression in endothelial cells results in oncogene induced senescence, which can be overcome by a secondary mutation. **a** Representative images showing endothelial cells that were left untreated or incubated with dox for 24 h to induce TC expression, then stained for DAPI (nuclei; blue), p16 (magenta), and FLAG (TC; yellow). Imaging was performed on a Zeiss fluorescence widefield microscope using a 40x objective. Scale bars = 25 μm. **b** Fluorescence intensity of p16 staining as shown in (**a**), to quantify its expression, n = 3. Statistical significance was determined with an unpaired t-test. **c** Representative images of GFP (TC) high, low, negative and uninduced populations stained for SA-β-gal activity, 4 days after addition of dox. **d** Frequency of cells positive for SA-β-gal activity in GFP high, GFP low, GFP-, and dox- endothelial cell populations. A minimum of 150 cells per condition were analysed, n = 3. Significance was calculated with a one-way ANOVA and Tukey’s multiple comparisons test. **e** Representative images showing the formation of GFP+ foci, whereby GFP is a marker for TC expressing endothelial cells, after 10 days exposure to dox. Scale bars = 200 μm, n = 3. **f** Fold change of TC high population between days 7 and 14 after nucleofection with control and *Cdkn2a* knockout guide RNA, n = 4. Statistical significance was calculated with a two-way ANOVA and Sidak’s multiple comparisons test. **g** Normalised cell number in *Cdkn2a* knockout endothelial cells, treated with dox or not, over 14 days, n = 4. Statistical significance was calculated with a two-way ANOVA and Sidak’s multiple comparisons test. All P-values are outlined in Supplementary Table 1. **h** Fold change of Ki67 expression within the TC high population between days 7 and 14 after addition of dox in comparison to uninduced endothelial cells, n = 4. Statistical significance was calculated with a two-way ANOVA and Sidak’s multiple comparisons test. **i** Representative images showing the formation of GFP+ foci, whereby GFP is a marker for TC expressing endothelial cells, after *Cdkn2a* knockout. Scale bars = 200 μm, n = 4. In all panels error bars show SEM, and *p < 0.05, **p < 0.01, ***p < 0.001, ****p < 0.0001.

HR-deficient cancer cells may undergo oncogene induced senescence (OIS), as the amount of DSBs overwhelms compensatory repair pathways^29^. Hence, we next investigated whether this was the case for TC-expressing endothelial cells in our model system. As senescent cells do not proliferate, this might explain the reduced cell growth observed for TC-expressing endothelial cells as shown above (Fig. 1). To explore this possibility, endothelial cells were exposed to dox for 24 h to induce TC expression, then p16 expression was determined by immunofluorescence, as this protein is a known positive regulator of senescence. Importantly, *CDKN2A*, which encodes p16, is the most common secondary mutation in EHE, and these tumours are often more aggressive^10^. Fluorescence microscopy revealed that dox induced TC-expressing endothelial cells had a significantly higher expression of p16 than untreated controls (Fig. 5a, b). Interestingly, some cells presented with a cytoplasmic expression of TC and a nuclear expression of p16, suggesting a transition where, before being shut down, TC is first translocated to the cytoplasm concomitant to p16 expression (Fig. 5a; white arrowhead).

We also assessed senescence-associated β-galactosidase activity (SA-β-gal) in endothelial cells expressing TC or not, another hallmark of senescent cells^31^. To this end, GFP sorted endothelial cells were cultured for 4 days and subjected to SA-β-gal staining (Fig. 5c, d). This revealed a higher proportion of cells with SA-β-gal activity in GFP high and low populations, compared to GFP^neg^ and untreated endothelial cells (Fig. 5d). Of note, the GFP high and low populations also took on the characteristic flat morphology of senescent cells, whereas GFP^neg^ and uninduced cells retained their endothelial morphology (Fig. 5c). Together, these data revealed that TC expression resulted in OIS in endothelial cells, following increased in p16 expression.

To study the long-term consequences of TC expression, endothelial cells were cultured for 4 weeks, with dox added every three days to maintain TC expression. After 10 days, small cell aggregates began to form, which were not present in the no dox cultures (Fig. 5e). These aggregates were GFP positive, suggesting sustained TC expression. Hence, we hypothesised that a subset of TC-expressing cells may bypass senescence by acquiring a secondary mutation. CDKN2A was shown to be the most common secondary mutation in EHE, conferring increased tumour aggressiveness^10^. As a proof of principle of the additional effect of secondary mutation, we investigated the effect of CRISPR/Cas9 *Cdkn2a* knockout on endothelial cell growth, expressing TC or not (Fig. 5f–i, Supplementary Fig. 9, 10). Here, flow cytometry revealed that the proportion of TC high cells increased significantly by day 14 in the *Cdkn2a* knockout condition, in contrast to the dox^+^ controls (Fig. 5f, Supplementary Fig. 9). Additionally, *Cdkn2a* knockout in dox treated cells resulted in a significant increase in cell number compared to control dox treated cells at all the time points tested (Fig. 5g, Supplementary Fig. 9, Supplementary Table 1) and the formation of very large GFP^+^ cell clusters (Fig. 5i). *Cdkn2a* knockout had little effect on untreated populations. Moreover, the number of Ki67+ TC high cells increased in all conditions between days 7 and 14, however this was only significant in *Cdkn2a* knockout populations (Fig. 5h, Supplementary Fig. 10). This provides evidence that a secondary, senescence-bypassing mutation is able to overcome the growth arrest imposed by TC expression.

### The interaction between TC and TEAD is required to sustain proliferation

**Fig. 6:**
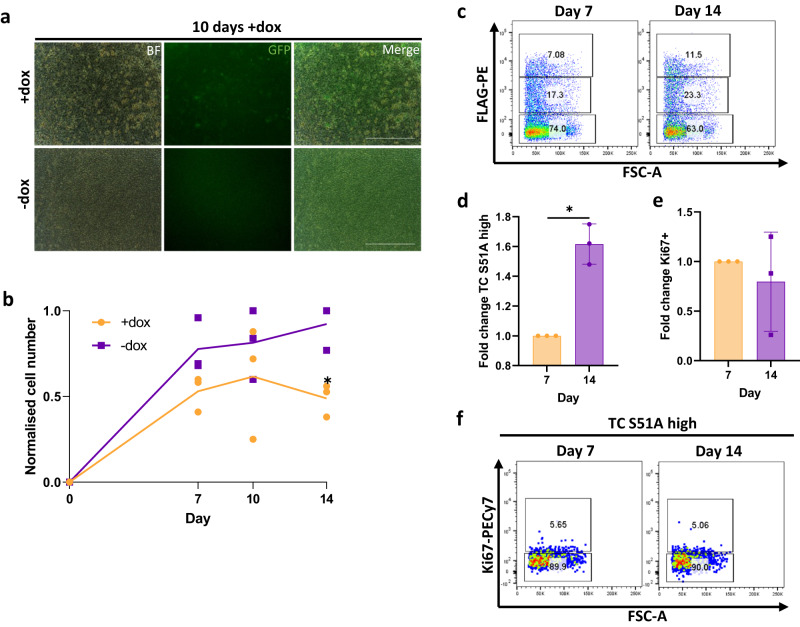
The interaction of TC and TEAD is required for maintenance of tumorigenesis. **a** Representative images showing endothelial cells treated with dox or not to induce TC S51A expression, whereby GFP is a marker for TC S51A, after 10 days exposure to dox. Scale bars=200μm, n = 3. **b** Normalised cell number in endothelial cells treated with dox or not to induce TC S51A expression, over 14 days, n = 3. Statistical significance was calculated with a two-way ANOVA and Sidak’s multiple comparisons test. **c** Representative flow cytometry plots showing TC S51A expression against FSC-A at day 7 and 14. **d** Fold change of TC S51A high population between days 7 and 14 after addition of dox in comparison to uninduced endothelial cells, n = 3. Statistical significance was calculated with a paired t-test. **e** Fold change of Ki67 expression within the TC S51A high population between days 7 and 14 after addition of dox in comparison to uninduced endothelial cells, n = 3. Statistical significance was calculated with a paired *t* test. **f** Representative flow cytometry plots showing Ki67 expression within the TC S51A high population against FSC-A at day 7 and 14. In all panels error bars show SEM, and *p < 0.05, **p < 0.01, ***p < 0.001, ****p < 0.0001.

Using a TC-S51A mutant, we showed above that the interaction between TC and TEAD was not essential for the generation of DSBs and hypertranscription upon TC initial expression in endothelial cells (Fig. 2, Supplementary Fig. 3, 7). However, many previous studies have highlighted that the interaction of TC with TEAD is important for tumorigenesis^11,14,15^. Therefore, we aimed to determine if the TC-TEAD interaction had a role in maintaining tumorigenesis in endothelial cells upon longer-term culture. Here, we generated endothelial cells and added dox to induced TC-S51A expression. Over the following 14 days, we analysed cell number and TC-S51A expression in comparison to untreated cells. Unlike TC-expressing endothelial cells, we did not observe GFP^+^ colony formation in TC-S51A expressing cultures after 10 days of dox induction (Fig. 6a). Moreover, TC-S51A expressing cultures remained at a lower cell number than untreated controls at all time points examined (Fig. 6b). This is in contrast to TC-expressing endothelial cells, which resulted in a comparable cell number to uninduced controls by day 14 (Fig. 5f–h). Similar to TC, the frequency of cells expressing TC-S51A did increase throughout the experiment (Fig. 6c, d). However, the number of FLAG high Ki67^+^ cells remained the same between days 7 and 14 (Fig. 6e, f). Overall, this shows that while the TC-TEAD interaction appears to be dispensable for DSB accumulation, it is required to sustain proliferation and tumour progression.

## Discussion

**Fig. 7:**
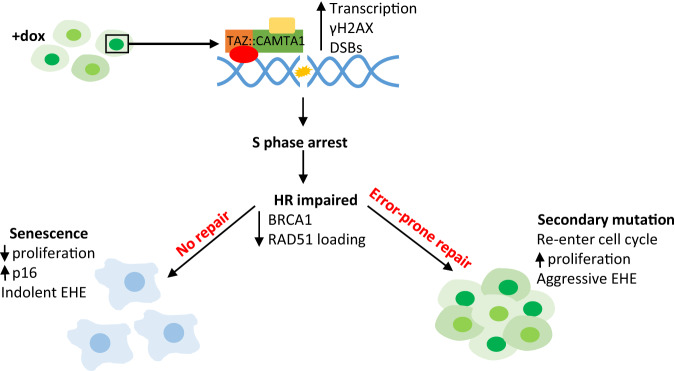
TC expression in endothelial cells results in senescence and genomic instability. Schematic showing how TC expression in endothelial cells results in hypertranscription, leading to the accumulation of DNA damage which in turn causes S phase arrest. As TC-expressing endothelial cells have impaired HR, as shown by reduced BRCA1 and RAD51 foci formation, many become senescent as the large amount of DNA damage is not repaired. These cells have increased p16 expression and remain arrested. In a subset of cells an error-prone DNA repair pathway will take over, leaving these vulnerable to acquiring growth-promoting secondary mutation.

Here, we describe an in vitro model for studying the immediate consequences of TC expression in primary endothelial cells, and how this contributes to EHE development. Using this model, we established that hypertranscription upon TC expression generates DSBs and causes cell cycle arrest (Fig. 7). Impairment of the HR pathway leaves these DSBs unresolved, leading to OIS in most cases. However, this also leaves TC-expressing endothelial cells susceptible to acquiring a growth-promoting secondary mutation. Previous studies have highlighted TC expression as being the initiating event in EHE development, however, these studies have used either contextually non-relevant cell lines or mouse models, which take a long time to generate tumours^5,11,14–16,41^. Using our model system, we provide a mechanistic explanation for previously unanswered questions about the unique clinical features of EHE within a contextually relevant, in vitro model.

EHE is described as having an unpredictable disease course; indolent tumours can switch to a highly aggressive phenotype^1,2^. We were able to replicate and provide mechanistic explanation for these clinical features. We show that upon TC expression, endothelial cells accumulate overwhelming DNA damage, initially causing increased p16 expression and cell cycle arrest. This later leads to SA-β-gal activity and a decreased growth rate, which are all hallmarks of OIS^31^. We therefore propose that the indolent phase of EHE is likely due to the onset of OIS in TC expressing endothelial cells. OIS also explains the low mutational burden of EHE tumours in the face of genomic instability, as growth arrest is imposed before any error-prone repair mechanism can take place and mutations passed on to daughter cells.

Our data suggest that TC-expression in endothelial cells alters cell cycle distribution compared to controls. TC expressing populations appear to be less sensitive to the double thymidine block, with a lower proportion of G1 phase and higher proportion of S-phase cells at 0 h compared to uninduced endothelial populations. A similar phenomenon is present in asynchronous populations. This presents premature entry into S-phase as a mechanism behind replication stress and DNA damage present in TC-expressing endothelial cells^18,42^. Subsequently, TC-expressing cells become arrested, evidenced by the accumulation of cells in late S/G2 phase at later time points after release of cell cycle block. Wild-type TAZ/YAP have been implicated in regulating S-phase entry, hence this may represent a function of the TAZ moiety in the transforming abilities of TAZ::CAMTA1^4,34^.

We also reveal that in TC-expressing endothelial cells, the combination of replication stress induced DNA damage and impaired HR contribute to genomic instability, a hallmark of cancer^17^. Moreover, secondary mutations are present in at least 55% of EHE tumours, and these cases tend to be more aggressive^10^. The most common of these secondary mutations affects *CDKN2A*, which encodes p16, a key positive regulator of senescence^10,31^. We were able to establish that *Cdkn2a* knockout in our model system allowed increased proliferation compared to *Cdkn2a* competent counterparts. This highlights the acquisition of a senescence-bypassing mutation as a mechanism behind the development of aggressive EHE. This is in line with data recently published, using a genetically engineer mouse model of TC expression and *Cdkn2a* deletion^43^.

Our model provides evidence that TC expressing endothelial cells have compromised DNA repair, specifically the HR pathway. This phenomenon is present in many cancers, with well-known examples including breast and ovarian tumours^44^. In these tumours, error-prone pathways take over, leading to accumulation of mutations^28^. Importantly, the discovery that HR is impaired in TC-expressing endothelial cells provides insight into potentially useful treatment options. Our data suggests that drugs targeting DNA repair, such as PARP or ATR inhibitors, may induce apoptosis preferentially in EHE tumour cells through synthetic lethality^45,46^.

Interestingly, our data highlights the striking similarities between the mechanism of action of TC and other fusion proteins found in sarcomas. Often, these fusion proteins dysregulate transcription by recruiting chromatin modifying complexes, as is seen for TC in EHE^16,47^. One of the best studied fusion proteins is EWS-FLI1, which is found in Ewing sarcoma. Here, EWS-FLI1 expression induces hypertranscription and DNA damage, leading to growth arrest^40,48^. Another commonality between TC and EWS-FLI1 is the induction of HR deficiency, while the tumours maintain a low mutational burden^40,47,49^. This is observed in Ewing sarcoma, but also in rhabdomyosarcoma (driven by PAX3/7 fusion proteins) and synovial sarcoma (SS18-SSX)^50,51^. Frequently, these fusion protein-defining sarcomas exhibit a loss of p16 which is associated with increased aggressiveness, suggesting a role for OIS^48,50,52–54^. Together, these findings could provide insight into broadly relevant treatment strategies for EHE. Examples include targeting chromatin modifiers, CDK4/6 inhibition and senolytics, which have proven efficacious in the other sarcomas mentioned above^47,55,56^. This is particularly important for rare cancers such as EHE, where studies are limited by sample size.

It would also be of interest to investigate the mechanisms behind HR-impairment in TC-expressing endothelial cells. Often, in other cancers, this is due to an inactivating mutation in a key HR protein, such as BRCA1/2, and represents the initiating event^28^. This cannot be the case in EHE as the DNA damage and growth arrest phenotype is evident immediately after TC expression, and there is no common HR inactivating mutation across tumour samples^10^. We hypothesise that TC may interfere with HR at the protein level, for example by inhibiting BRCA1 repair complex formation. Similar phenomena have been observed in Ewing sarcoma, where BRCA1 remains associated with the transcriptional machinery despite DSBs, and with oncogenic RAS, which induces BRCA1 chromatin dissociation^29,40^. Further study will be required to investigate how TC interferes with HR repair mechanisms.

Previous studies have demonstrated the ability of TC to interact with TEAD as vital to its transforming ability^11,14,15^. Here, we show that the induction of DSBs is independent of the TC-TEAD interaction. We propose that this is due to the replication stress induced by increased transcription being primarily mediated by the CAMTA1 moiety and TAZ interacting with other transcription factors^7,8^. Previous reports suggest that CAMTA1 can recruit the ATAC complex for chromatin modification, which would permit the large transcriptomic changes required for induction of replication stress^16^. Despite this, our model does show significant enrichment of canonical TAZ/YAP gene sets upon TC expression, in agreement with current studies. It is also evident that the TC-TEAD interaction is still crucial to EHE progression, as in longer term cultures TC-S51A expressing endothelial cells were unable to overcome growth arrest. In many published studies, transformed cell lines were used to study the effects of TC and TC-S51A^14–16^. We propose that using already transformed cell lines mimics the effect of a secondary mutation, allowing tumorigenesis as soon as TC is expressed.

To summarise, we present here a model for generating endothelial cells from mESCs, which can be used to study EHE by inducing TC expression. This inducible in vitro model is very powerful as it allows generating large quantities of cells for analysing the immediate consequences of TC expression. Our model reveals that TC expression rapidly results in OIS and genomic instability and that these processes may explain the clinical features of EHE. Understanding the molecular mechanism by which the oncogenic fusion protein TC promotes tumour progression will help devise novel treatments for EHE patients.

## Methods

### Maintenance of mouse embryonic stem cells

The mESCs contain an inserted construct into the HPRT locus whereby TC or TC S51A expression is induced upon the addition of doxycycline (dox) as previously described^57^. Both TC and TC S51A sequences contain an N-terminal double FLAG epitope and is followed by an IRES-GFP reporter cassette. In all experiments that required TC or TC S51A protein expression, dox was added to the culture media at a final concentration of 1 μg/ml. Parallel control experiments were set without dox (uninduced). mESCs were expanded and maintained in DMEM-ES medium. This comprised of Dulbecco’s Modified Eagle Medium (DMEM; Sigma) supplemented with 15% foetal calf serum (FCS), 2 mM L-glutamine (Gibco), 50 U/ml penicillin-streptomycin (Sigma), 1% LIF conditioned media, and 0.15 mM Monothioglycerol (MTG; Sigma). Cells were maintained on a monolayer of Mitomycin C inactivated mouse embryonic fibroblasts (MEFs) until differentiation. mESCs were cultured at 37 °C with 5 % CO_2_ in a humidified incubator.

### Maintenance of HEK293T cells

HEK293T cells were cultured and transfected as previously described^58^. Briefly, 50% confluent cells in T75 flasks were transfected by the polyethyleneimine (PEI) method with 5 μg of Flag-tagged TAZ, TAZ::CAMTA1 and TAZ-CAMTA-1 S51A open reading frames cloned into pcDNA3.1 expressing vectors. Cells were cultured for 48 h prior cell pellet collection.

### Generation and maintenance of mESC-derived endothelial cells

To initiate the endothelial differentiation process, mESCs were passaged twice onto 0.1% gelatine coated plates without MEFs. The cells were first passaged in DMEM-ES, then in IMDM-ES. IMDM-ES contains IMDM supplemented with 15% FCS, 2 mM L-glutamine, 50 U/ml penicillin-streptomycin, 1% LIF conditioned media, and 0.15 mM MTG. Next EBs were generated by harvesting mESCs and transferring IMDM supplemented with 15% FCS, 2 mM L-glutamine, 50 U/ml penicillin-streptomycin, 0.5 ng/ml ascorbic acid (Sigma), 180 µg/ml transferrin (Sigma), 0.45 mM MTG and 5 ng/ml human vascular endothelial growth factor (hVEGF; Peprotech). EBs were cultured in non-tissue culture treated 10 cm dishes. From this point on, cells were maintained in low oxygen (5% O_2_) conditions, at 37 °C with 5% CO_2_ in a humidified incubator. At day 5 EBs were subject to cell sorting to isolate the TIE2^+^FLK1^+^ population. After sorting, cells were cultured in endothelial cell medium, which contains IMDM with 10% FCS, 2 mM L-glutamine, 50 U/ml penicillin-streptomycin, 180 µg/ml transferrin, 0.45 mM MTG, 0.25 ng/ml ascorbic acid, 15% D4T conditioned medium, 5 ng/ml murine basic fibroblast growth factor (bFGF; Peprotech) and 5 ng/ml hVEGF. Cells were maintained on Matrigel (Corning) diluted to a protein concentration of 5.5 mg/ml in IMDM. Endothelial cell medium was replaced every 2–3 days and supplemented with dox as appropriate.

### Flow cytometry

For analysis of extracellular markers, cells were dissociated with TrypLE Express (Gibco) at the indicated time points. Cells were washed using FACS wash (1X PBS 10% FCS) to create single cell suspensions. Staining was carried out for 30 min at 4 °C, using the antibodies listed in Supplementary Table 2. Cells were also analysed for GFP as a marker for TC or TC S51A expression, depending on the cell line used. After staining, cells were washed and resuspended in FACS wash prior to analysis. For intracellular analysis, cell pellets were resuspended in 2% paraformaldehyde (PFA; Alfa Aesar) in 1X PBS for 20 min at room temperature. Cell were then permeabilized using 0.1% saponin in FACS wash for 15 min at room temperature. Fluorophore pre-conjugated or unconjugated primary antibodies were then added to samples at the appropriate concentrations, then left to stain for 30 min at 4 °C. If used, fluorophore pre-conjugated secondary antibodies were then added and incubated for 30 min at 4 °C. Afterwards, cell samples were washed then resuspended in 0.1% saponin in FACS wash for analysis. All analysis was performed using an LSR Fortessa cytometer (Becton Dickinson).

### Cell sorting

For sorting the TIE2^+^FLK1^+^ cell population, EBs were harvested and allowed to settle under gravity, before dissociation using TrypLE Express. Cells were then resuspended in IMDM 10% FCS and passed through a 50 μm Filcon (Becton Dickinson), prior to staining with TIE2 and FLK1 antibodies (Supplementary Table 2), and Hoechst 33258 for 1 h at 4 °C. Cell samples were vortexed after 30 min to prevent clumping. After staining, samples were washed and resuspended in IMDM 10% FCS, and sorted using the FACS Aria or Influx cell sorters (Becton Dickinson) at 4 °C. After sorting, TIE2^+^FLK1^+^ cells were collected and re-plated in conditions for endothelial cell culture, as described above.

### Cell cycle analysis

For cell cycle analysis, cells were synchronised at the G1/S phase boundary using a double thymidine block. Here, endothelial cells were treated with 2 mM thymidine (Sigma) in cell culture grade H_2_O for 18 h then released into fresh media for 8 h. Endothelial cells were then subject to a second block using 2 mM thymidine again for 16 h, then released into fresh media. At this point, 20 μM 5-ethynyl-2’-deoxyuridine (EdU) in DMSO was added, then samples were harvested every 2 h for 10 h in total. The cells were then fixed in 4% paraformaldehyde for 15 min and stored in 1X PBS overnight at 4˚C. The next day, staining and the click chemistry reaction to detect EdU incorporation was carried out using the Click-iT Plus EdU Alexa Fluor 647 Flow Cytometry Assay Kit (Invitrogen), as per manufacturer’s instructions. Cells were then stained with Hoechst 33258 to analyse DNA content, and an anti-FLAG tag antibody (Supplementary Table 2) to determine TC expression level.

### RNA sequencing and analysis

To collect samples for RNA sequencing, mESC-derived endothelial cells were cultured for 10 days after TIE2^+^ FLK1^+^ antibody sorting, before inducing TC expression or not with dox. 24 h later, endothelial cells were harvested and sorted by flow cytometry into four populations: TC high, TC low, TC negative, and uninduced. TC expression levels were defined by GFP expression. After sorting, cell pellets were resuspended in RLT lysis buffer from the RNeasy Mini Kit (Qiagen) and stored at −80 °C until sufficient samples for three biological repeats had been collected. RNA extraction was then performed on all samples at the same time, using the RNeasy Mini Kit, as per manufacturer’s instructions. RNA concentration was quantified using the Qubit RNA BR Assay Kit (Invitrogen) and Qubit 3 fluorometer (Invitrogen). Sequencing was performed by the Genomic Technologies Core Facility at the University of Manchester, using the Illumina HiSeq 4000 system. Unmapped paired-end sequences were assessed by FastQC, then sequence adaptors were removed and reads trimmed using Trimmomatic_0.36. The reads were then aligned to the mm10 reference mouse genome, and gene counts were calculated using annotation from GENCODE M25 using STAR_2.7.2b. Normalisation, principal component analysis, and differential gene expression was calculated in DESeq2_1.20.0 using default settings. This bioinformatics analysis was performed by the Bioinformatics Core Facility at the University of Manchester. Accession number for RNA-seq data: E-MTAB-11882 on the EMBL-EBI ArrayExpress database.

### Comet assay

A neutral comet assay was performed on day 10 cultures of mESC-derived endothelial cells treated with dox or not for 24 h to induce either TC or TC S51A expression. For a positive control, endothelial cells were treated with 30 μM H_2_O_2_ for 4 h. The neutral comet assay was performed using the Comet SCGE Assay Kit (Enzo Lifesciences) as per manufactures instructions. Phase contrast imaging was undertaken using a Leica DMI 3000B inverted microscope fitted with an N PLAN 20x/0.35 NA PH1 objective (PH1 filter) and a DFC310FX camera. Acquisition was controlled by the Leica LAS X software.

### Immunofluorescence

Prior to immunofluorescent staining, endothelial cells were grown on 12 well chamber slides (Ibidi), coated with phenol red free matrigel (Corning) diluted to a protein concentration of 5.5 mg/ml in 1X PBS. TC or TC S51A expression was induced with dox at the indicated time points prior staining. Where indicated, cells were treated with 2 mM hydroxyurea for 2 h or 30μM H_2_O_2_ for 4 h at 37 °C, prior to fixation. Culture media was removed then cells were washed with 1X PBS, before fixation using 2% paraformaldehyde in 1X PBS for 20 min at room temperature. Where indicated for R-loops staining, samples were treated with 1:200 RNaseH diluted in blocking and permeabilisation buffer (1X PBS, 5% goat serum, 0.3% Triton X-100) for 1 h at 37 °C after fixation. Cells were washed with 1X PBS, then blocking and permeabilisation buffer was added to each well and incubated for 1 h at room temperature. The blocking buffer was then removed and replaced with the primary antibodies (Supplementary Table 2) diluted in antibody dilution buffer (1X PBS, 1% BSA, 0.3% Triton X-100), and incubated for either 1 h at room temperature or overnight at 4 °C (BRCA1 antibody only). Next, each well was washed with 1X PBS, before incubation of the secondary antibodies (Supplementary Table 2) diluted in antibody dilution buffer for 1 h at room temperature, protected from light. Cells were washed again with 1X PBS before mounting using ProLong Diamond Antifade Mounting with DAPI (Invitrogen). Slides were then left to cure overnight at room temperature, protected from light. For long term storage, slides were kept at 4 °C in the dark. Imaging was carried out using the Zeiss Axio Imager M2 upright fluorescent microscope fitted with a Photometrics CoolSNAP HQ2 camera, using either a 40x/0.75 NA dry or 63x/1.3 NA oil immersion objective, as indicated. Acquisition was controlled by the Micro-Manager 2.0 software. DAPI staining was used to define nuclei and set masks in ImageJ (v2.0.0) for analysis of nuclear fluorescence intensity or foci positivity. To determine the number of foci/nuclei, the brightness/contrast parameters in ImageJ were kept consistent between images within each experiment and counted manually.

### Immunoprecipitation

Cell lysis and immunoprecipitation were carried out as previously described^59^ with modifications. Cells were washed twice with 1X PBS and pelleted down at 400xg for 5 min. Cell were lysed in 10 times the volume of the dry cell pellet in an IP buffer [50 mM Tris (pH 8.0), 150 mM NaCl, 1% NP-40, 0.5 % Sodium deoxycholate, 1% protease inhibitor cocktail (Sigma)], supplemented with 250 U/mL of Pierce’s Universal Nuclease. After 1 h of incubation at 4 C, the lysed was cleared by centrifugation at 20,000 × *g* and total protein concentration was estimated by using Protein Assay Dye Reagent (Bio-Rad #5000006). Immunoprecipitation of Flag-tagged proteins was carried out from 500 μg of total protein using 10 μL of FlagM2 magnetic beads (Sigma, M8823) and the DynaMag magnet (Life Technology). Beads-isolated immunocomplexes were washed 4 times with IP buffer and eluted with 2xLDS buffer (Invitrogen) supplemented with 200 mM DTT (Sigma). Samples were heated 5 min at 95 C ready for protein detection. Protein electrophoresis and western blots were carried out using the XCell SureLock mini system, the Novex® NuPAGE® SDS-PAGE Gel System (Life Technology), the Trans Blot Turbo system (Bio-Rad), and a Multicolour Broad Range Protein Ladder (Thermo Scientific, MAN0011774) under manufacturer recommendations. Nitrocellulose (GE Health Care) was used as a membrane for protein transfer and Ponceau S stain (Sigma) to assess protein transfer efficiency. TEAD proteins were detected by using an anti-pan specific TEAD antibody (Cell Signalling Technology, 13295S) at 1:3,000 dilution. Flag-tagged expressed proteins were detected by using a rabbit monoclonal anti Flag at 1:10,000 (Sigma, F7425). Secondary goat antibody anti rabbit IgG conjugated with HRP (Abcam Ab 97051) at 1:20,000 dilution, ECL prime Western blotting system and film exposure (Amersham) were used to visualise signal.

### Senescence-associated beta-galactosidase (SA-β-gal) detection

Prior to SA-β-gal detection, mESC-derived endothelial cells were treated with dox or not for 24 h prior to GFP cell sorting. Cells were then cultured for a further 4 days. The cells were then fixed for 15 min and stained using the Senescence Detection Kit (Abcam), as per manufacturer’s instructions. Cells were incubated in the staining solution overnight in a humidified incubator at 37 °C. The plate was placed in a ziplock bag during the overnight incubation, so as the incubator CO_2_ levels did not affect colour development. Phase contrast imaging was undertaken using a Leica DMI 3000B inverted microscope fitted with an N PLAN 20x/0.35 NA PH1 objective (PH1 filter) and a DFC310FX camera. Acquisition was controlled by the Leica LAS X software.

### CRISPR/Cas9 mediated gene knockout

For deletion of *Cdkn2a*, three different crRNA sequences were used and are outlined in Supplementary Table 3 (Integrated DNA Technologies). RNA duplex was formed by combining crRNA, Alt-R tracrRNA and Duplex Buffer (all Integrated DNA Technologies) to a final concentration of 50 μM, then heating at 95 °C for 5 min before cooling to room temperature. To form the RNP complex, RNA duplex was then added to Cas9 enzyme to a final concentration of 25 μM and 24.4 μM respectively and left at room temperature for 20 min. Nucleofection was performed using the Neon Transfection System (Invitrogen), as per manufacturer’s instructions. Endothelial cells were harvested and counted then resuspended in Buffer R. The RNP complex was then mixed with the cell suspension, and an electroporation protocol of 3 × 10 ms pulses at 1400 V was used. Cells were then resuspended in endothelial cell media supplemented with dox or not, then re-plated at 100,000 cells/well in a 12 well plate.

### RNA imaging

Imaging of nascent RNA was performed using the Click-iT RNA Alexa Fluor 594 imaging kit (Invitrogen). Cells were incubated with 1 mM 5-ethynyl uridine (5-EU) in DMSO for 1 h prior to fixation. Where indicated, samples were treated with 0.2 mg/ml RNaseA solution (Promega) diluted in blocking buffer for 1 h at 37 °C after fixation, as a negative control. Staining was conducted as per manufacturer’s instructions. Imaging was carried out using the Zeiss Axio Imager M2 upright fluorescent microscope fitted with a Photometrics CoolSNAP HQ2 camera, using a 63x/1.3 NA oil immersion objective. Acquisition was controlled by the Micro-Manager 2.0 software.

### Statistics and reproducibility

These are described for each experiment in the corresponding figure legends.

### Reporting summary

Further information on research design is available in the Nature Portfolio Reporting Summary linked to this article.

### Supplementary information

Supplementary Information

Description of Additional Supplementary Files

Supplementary Data

Reporting Summary

## Data Availability

Accession number for RNA-seq data: E-MTAB-11882 on the EMBL-EBI ArrayExpress database. All source data behind all graphs in the paper can be found in the file named Supplementary Data.
