# Supplementary material for: The oncogenic fusion protein TAZ::CAMTA1 promotes genomic instability and senescence through hypertranscription
Source: Commun Biol. 2023 Nov 18;6:1174. doi: 10.1038/s42003-023-05540-4 (PMC10657451; doi:10.1038/s42003-023-05540-4)
Supplement: Supplementary file 1 — Supplementary Information [file 42003_2023_5540_MOESM1_ESM.pdf]

Supplementary Figure 1

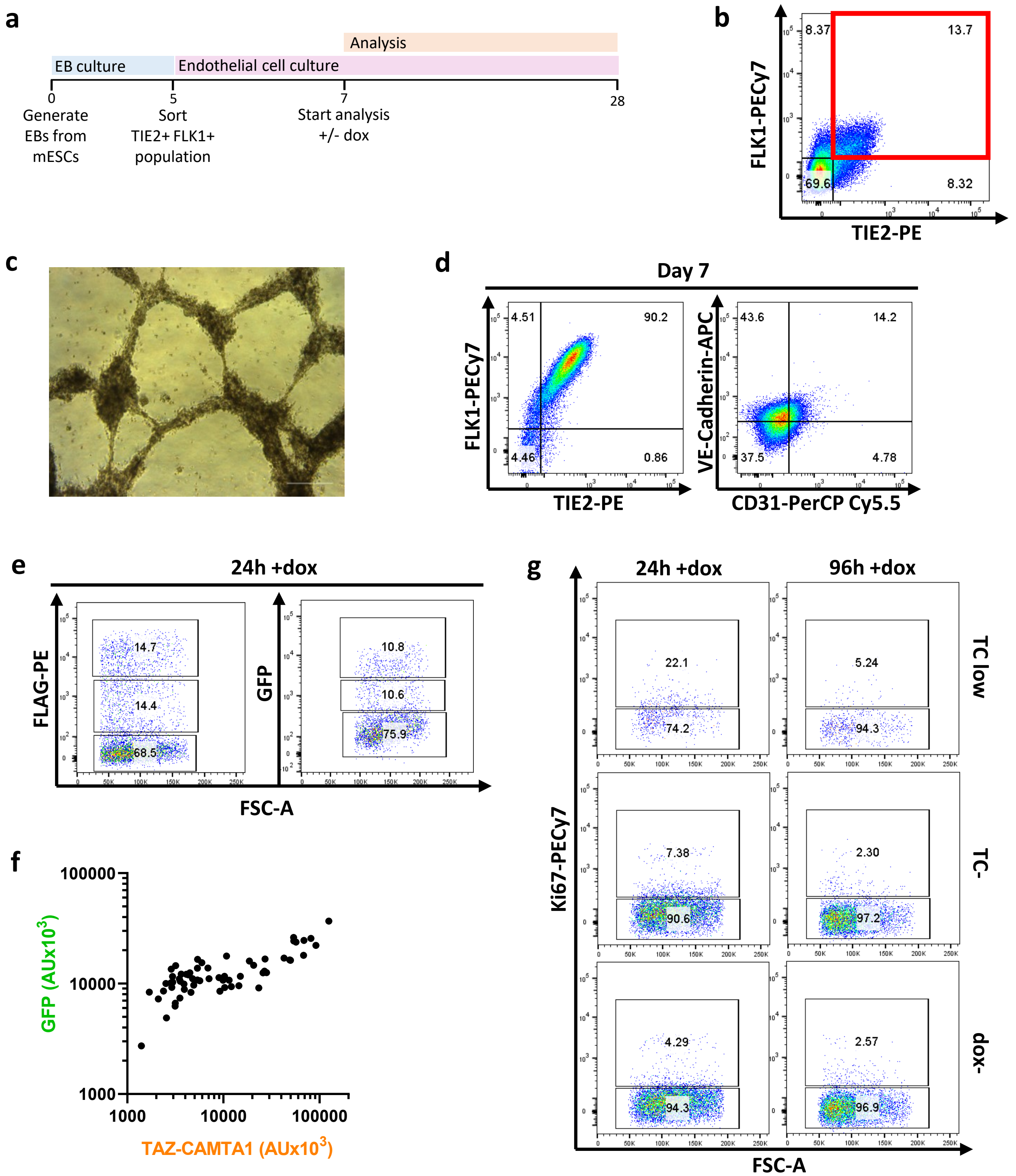

Supplementary Figure 1. A model for generating endothelial cells with inducible TC expression.

a Differentiation timeline for the production of endothelial cells from mESCs, by sorting a progenitor population from EBs.

b Representative flow cytometry plot showing the TIE2+ FLK1+ population sorted from day 5 EBs for endothelial cell differentiation.

c Representative image showing tube-like formation ability of endothelial cells in Matrigel plug. Scale bar=100μm.

d Representative flow cytometry plots showing the expression of four endothelial cell markers (FLK1, TIE2, CD31, VE-Cadherin) in endothelial cells 7 days after sorting.

e Representative flow cytometry plots showing FLAG and GFP expression against FSC-A, both of which are markers of TC.

f Positive correlation between TC expression, measured using the FLAG epitope, and GFP expression, which is expressed via an IRES upon dox induction for tracking induced cells.

g Representative flow cytometry plots showing the percentage of Ki67+ endothelial cells within TC low, neg, and uninduced populations, at 24h and 96h after dox induction.

h Representative flow cytometry plots showing EdU incorporation against Hoechst staining in TC high, TC low, TC- and uninduced endothelial cell populations when not subject to cell cycle synchronisation.

Supplementary Figure 2

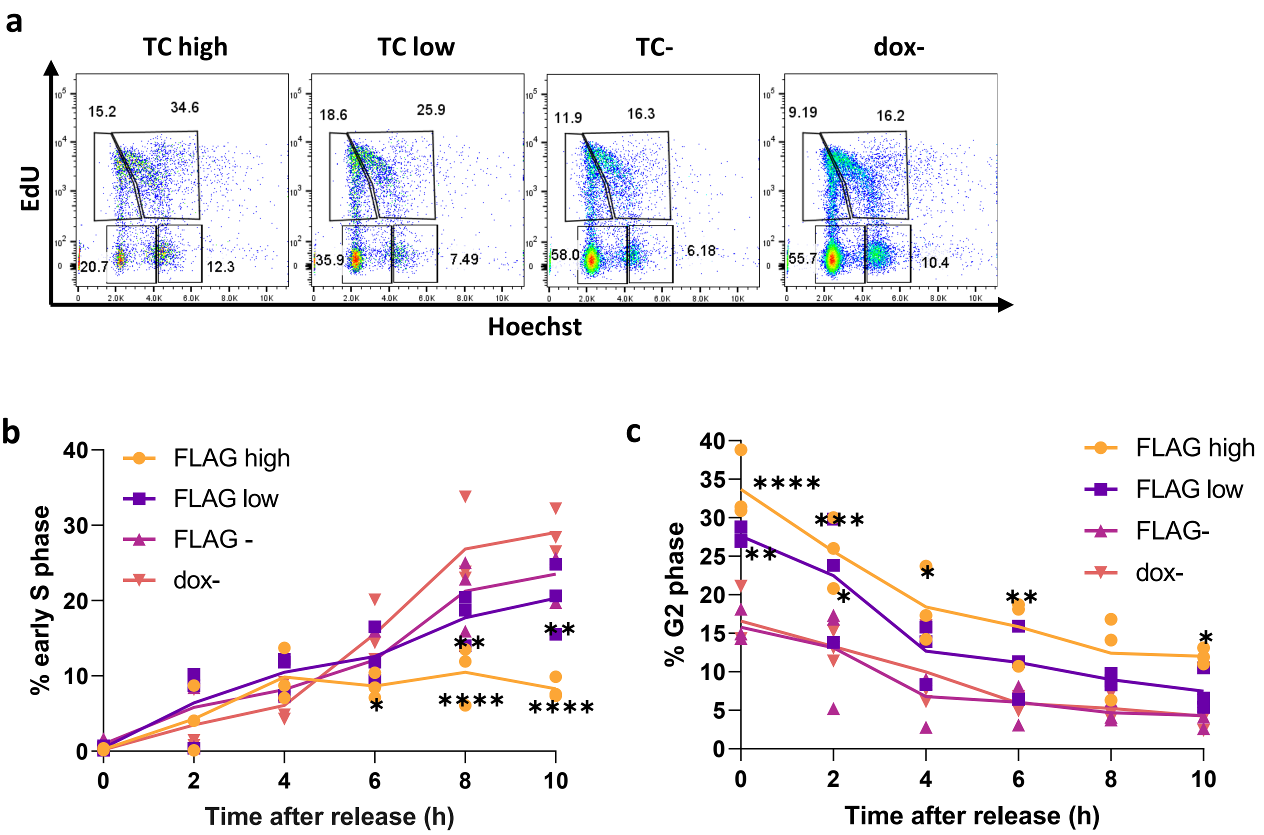

Supplementary Figure 2. TC expression causes endothelial cell cycle arrest.

a Representative flow cytometry plots showing EdU incorporation against Hoechst staining in TC high, TC low, TC- and uninduced endothelial cell populations when not subject to cell cycle synchronisation.

b Proportion of cells in early S phase over 10h following release from thymidine block, in TC high, TC low, TC- and uninduced endothelial cell populations, n=3.

c as **b** but for proportion of cells in G2 phase, n=3. Statistical significance was determined by two-way ANOVA with Dunnett's multiple comparisons test. In all panels error bars show SEM, and *p<0.05, **p<0.01, ***p<0.001, ****p<0.0001.

Supplementary Figure 3

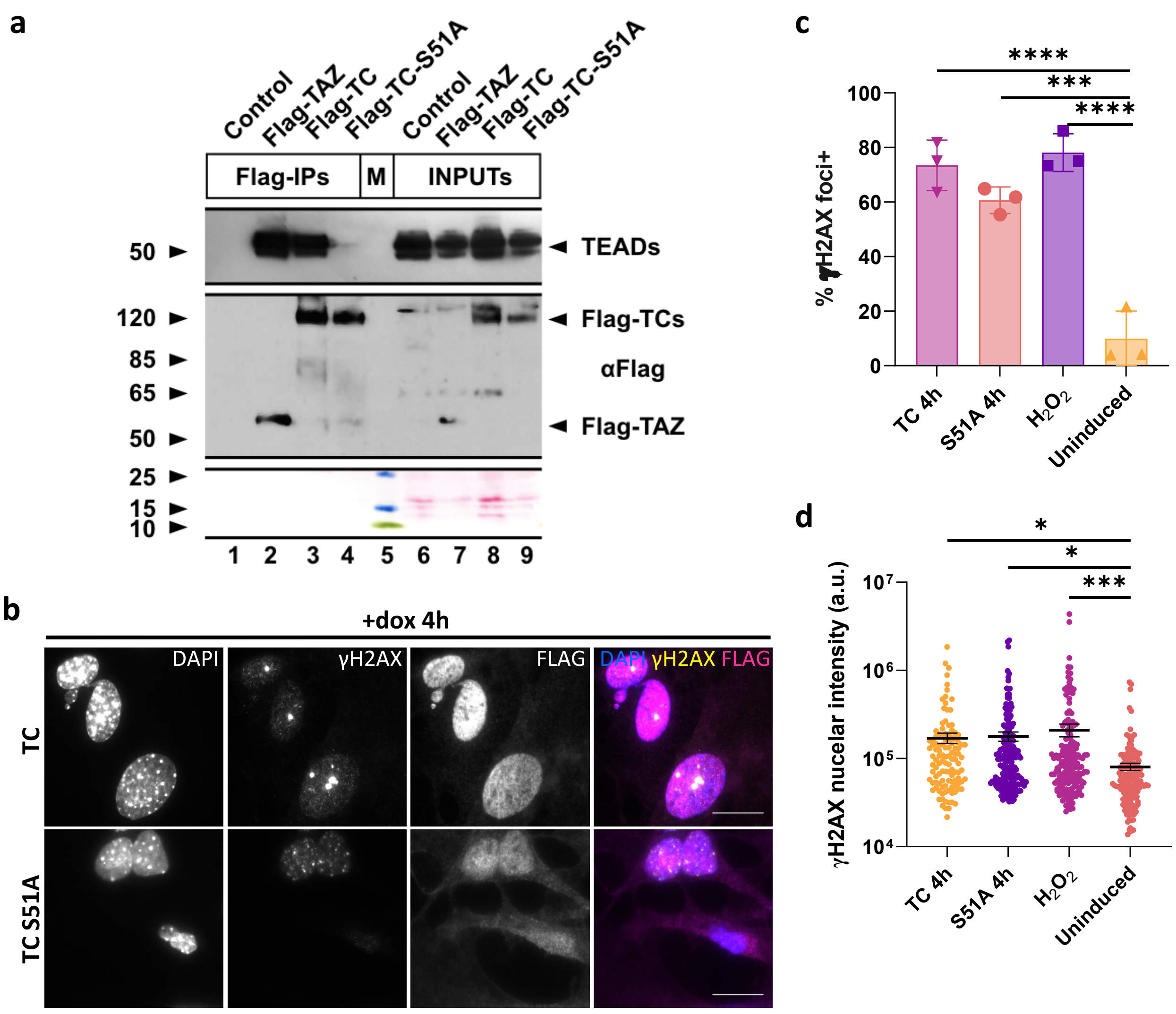

Supplementary Figure 3. TC causes endothelial cells to accumulate DNA double strand breaks immediately upon its expression

a Co-immunoprecipitation of exogenously expressed Flag-tagged TAZ, TC and TC-S51A proteins, and TEAD proteins (endogenous) in HEK293 cells. INPUT represents 10% of the total lysed used for the IP.

b Representative images showing endothelial cells treated with dox for 4h to induce TC expression. Cells treated with 30µM H_2O_2 for 4h were used as a positive control. Cells were stained with DAPI (nuclei; blue), FLAG antibody (TC; magenta), and γH2AX antibody (phospho-H2AX; yellow). All imaging was performed on a Zeiss fluorescence widefield microscope using a 63x oil immersion objective. Scale bars=20µm.

c The frequency of endothelial cells positive for γH2AX foci from the imaging experiments as presented in **b.** Cells with >10 foci were considered positive. Significance was calculated by one-way ANOVA and Dunnett's multiple comparisons test.

d Nuclear fluorescence intensity of γH2AX staining in cells from the imaging experiments as presented in **b.** Significance was calculated by one-way ANOVA and Dunnett's multiple comparisons test. In all imaging experiments, at least 150 cells per condition were analysed, n=3. In all panels error bars show SEM, and *$p<0.05$, **$p<0.01$, ***$p<0.001$, ****$p<0.0001$.

Supplementary Figure 4

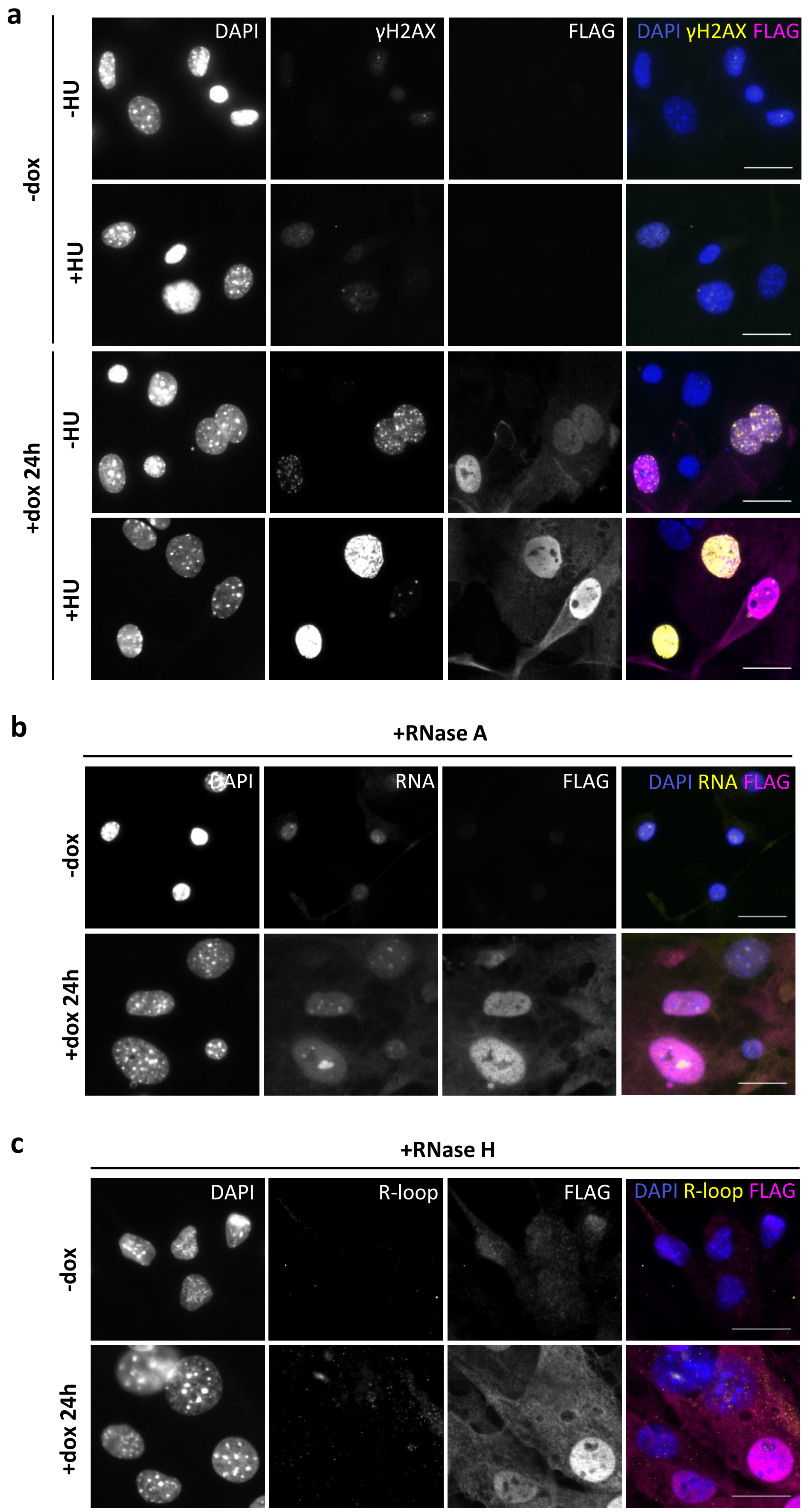

Supplementary Figure 4. TC expression mediates hypertranscription and replication stress in endothelial cells.

a Representative images showing endothelial cells treated with or without dox for 24h to induce TC expression, alongside hydroxyurea (HU) or not to induce replication stress. Cells were stained with DAPI (nuclei; blue), FLAG antibody (TC; magenta), and γH2AX antibody (phospho-H2AX; yellow).

b Representative images showing nascent RNA staining in endothelial cells incubated with 5-EU for 1h, with or without dox for 24h to induce TC expression. Cells were treated with RNaseA prior to staining as a negative control. Cells were stained for 5-EU (nascent RNA; yellow), DAPI (nuclei; blue) and FLAG (TC; magenta).

c Representative images showing S9.6 antibody staining to visualise R-loops in endothelial cells treated with or without dox for 24h to induce TC expression. Cells were treated with RNaseH prior to staining as a negative control. Cells were stained for R-loops (yellow), DAPI (nuclei; blue) and FLAG (TC; magenta).

All imaging was performed on a Zeiss fluorescence widefield microscope using a 63x oil immersion objective. Scale bars=20μm in all panels. In all imaging experiments, at least 150 cells per condition were analysed, n=3.

Supplementary Figure 5

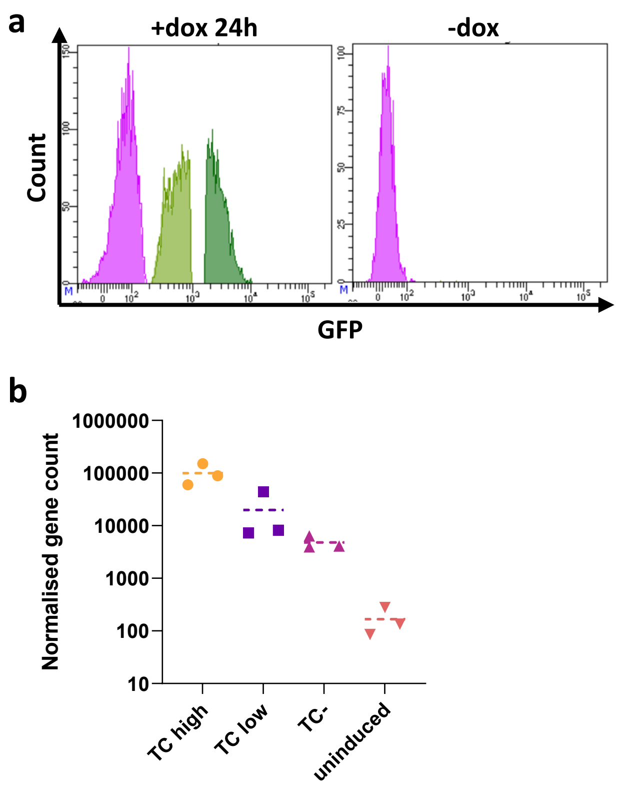

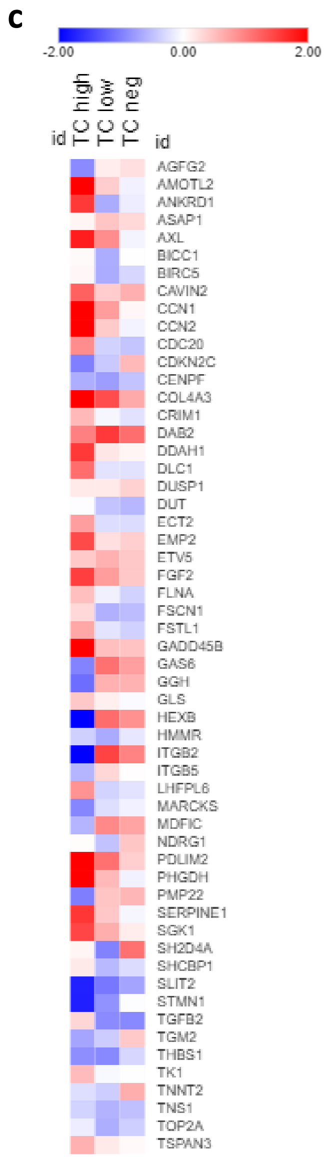

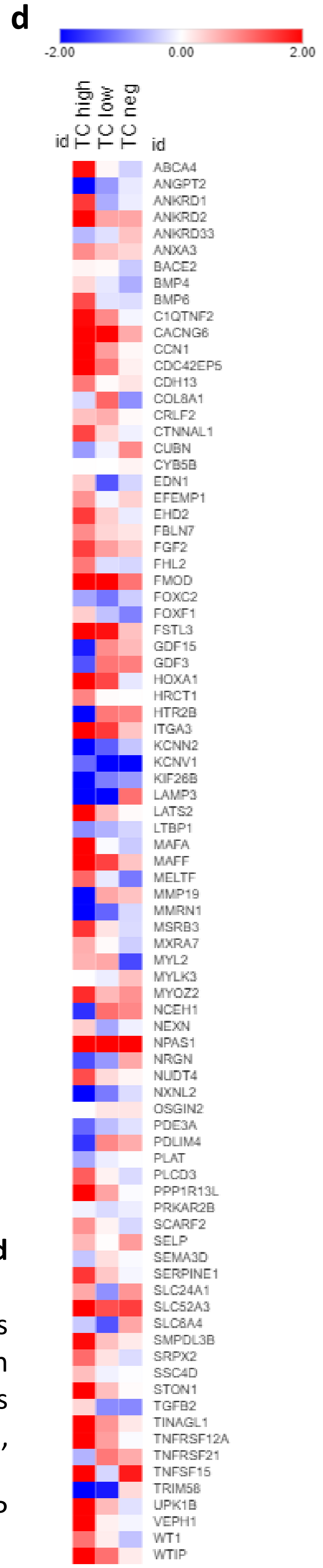

Supplementary Figure 5. RNA-seq analysis reveals enrichment for EHE and TAZ/YAP targets in TC expressing endothelial cells.

a Representative flow cytometry histograms showing the populations sorted from day 11 endothelial cells for RNA sequencing samples, based on GFP expression as a marker for TC expression. Dox was added or not to cells 24 hours prior, and then sorted into GFP- (purple), GFP low (light green), and GFP high (dark green) samples.

b TC RNA expression in each of the four samples sorted based on GFP expression in **a.**

c Heatmap showing log2 fold change in RNA expression of Cordenonsi YAP conserved signature gene set when comparing TC high, TC low and TC- endothelial cell populations to uninduced controls.

d Heatmap showing log2 fold change in RNA expression of Seavey Epithelioid Haemangioendothelioma gene set when comparing TC high, TC low and TC- endothelial cell populations to uninduced controls.

Supplementary Figure 6

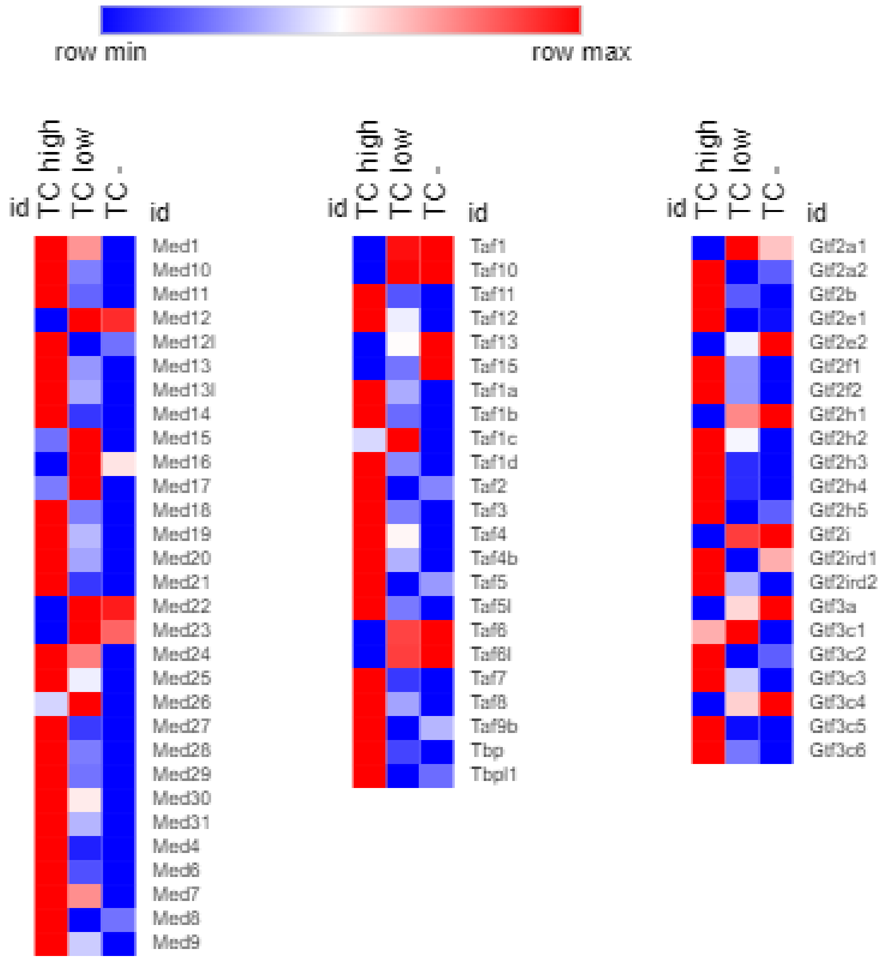

Supplementary Figure 6. RNA-seq analysis reveals increased expression of genes associated with active transcription mediated by polymerase II in TC expressing endothelial cells.
Heatmap showing log2 fold change in RNA expression of mediator subunit (Med) genes, TATA-binding protein (TBP) and TBP-associated factor (Taf) genes and general transcription factor (Gtf) genes when comparing TC high, TC low and TC- endothelial cell populations to uninduced controls.

Supplementary Figure 7

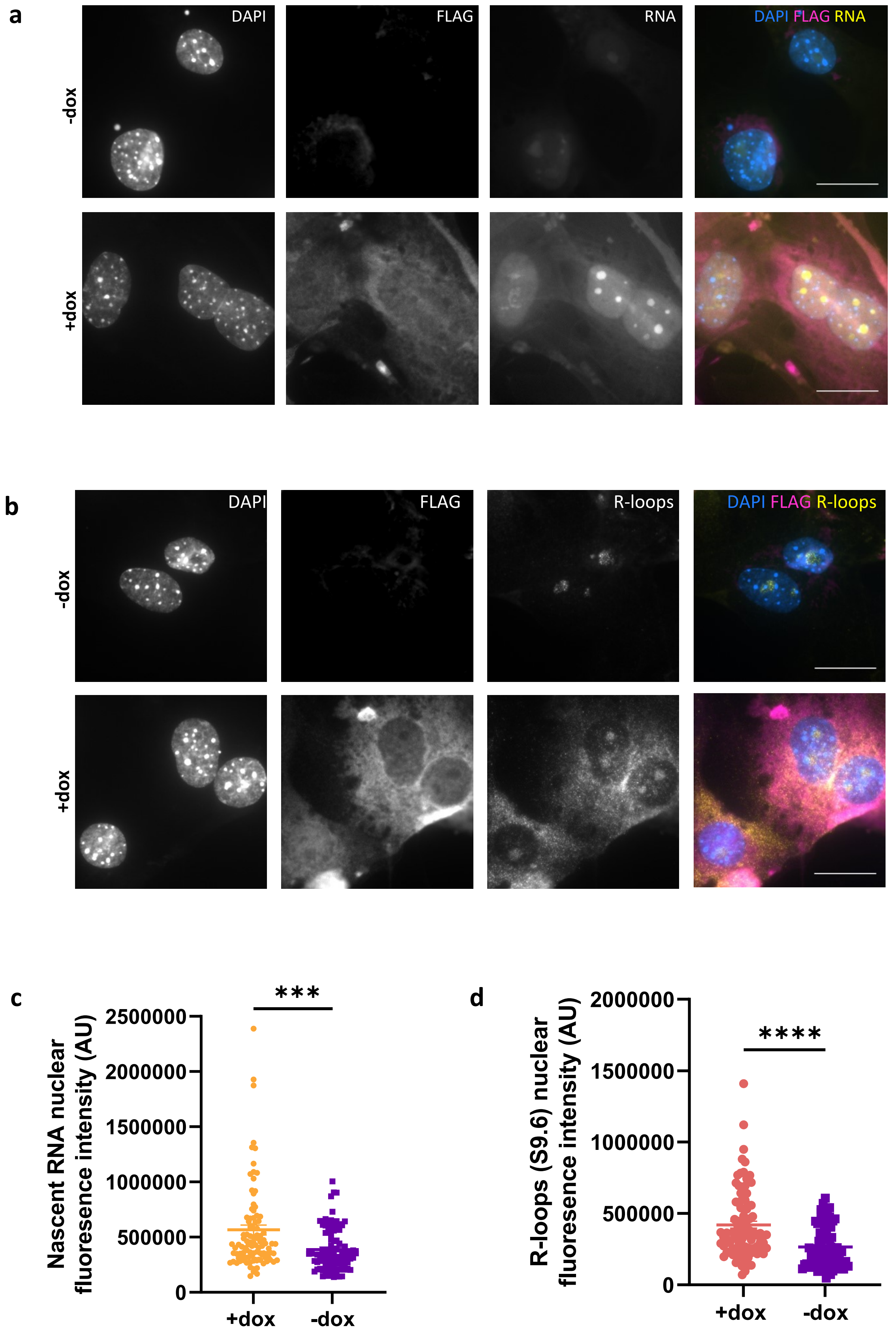

Supplementary Figure 7. TC-S51A expression mediates hypertranscription and replication stress in endothelial cells.

a Representative images showing nascent RNA staining after endothelial cells were incubated with 5-EU for 1h, and treated with or without dox for 24h to induce TC-S51A expression. Cells were stained for 5-EU (nascent RNA; yellow), DAPI (nuclei; blue) and FLAG (TC; magenta).

b Representative images showing S9.6 antibody staining to visualise R-loops in endothelial cells treated with or without dox for 24h to induce TC-S51A expression. Cells were stained for R-loops (yellow), DAPI (nuclei; blue) and FLAG (TC; magenta).

c Fluorescence intensity of nuclear 5-EU staining as shown in **a**, to quantify nascent RNA in endothelial samples treated with dox for 24h or not.

d Fluorescence intensity of nuclear S9.6 antibody staining as shown in **b**, to quantify the presence of R-loops in endothelial samples treated with dox for 24h or not.

Imaging was performed on a Zeiss fluorescence widefield microscope using a 63x oil immersion objective. Scale bars=20μm. In all imaging experiments, at least 145 cells per condition were analysed. Statistical significance was determined with a one-way ANOVA and Sidak’s multiple comparisons test, n=3. In all panels error bars show SEM, and *p<0.05, **p<0.01, ***p<0.001, ****p<0.0001.

Supplementary Figure 8

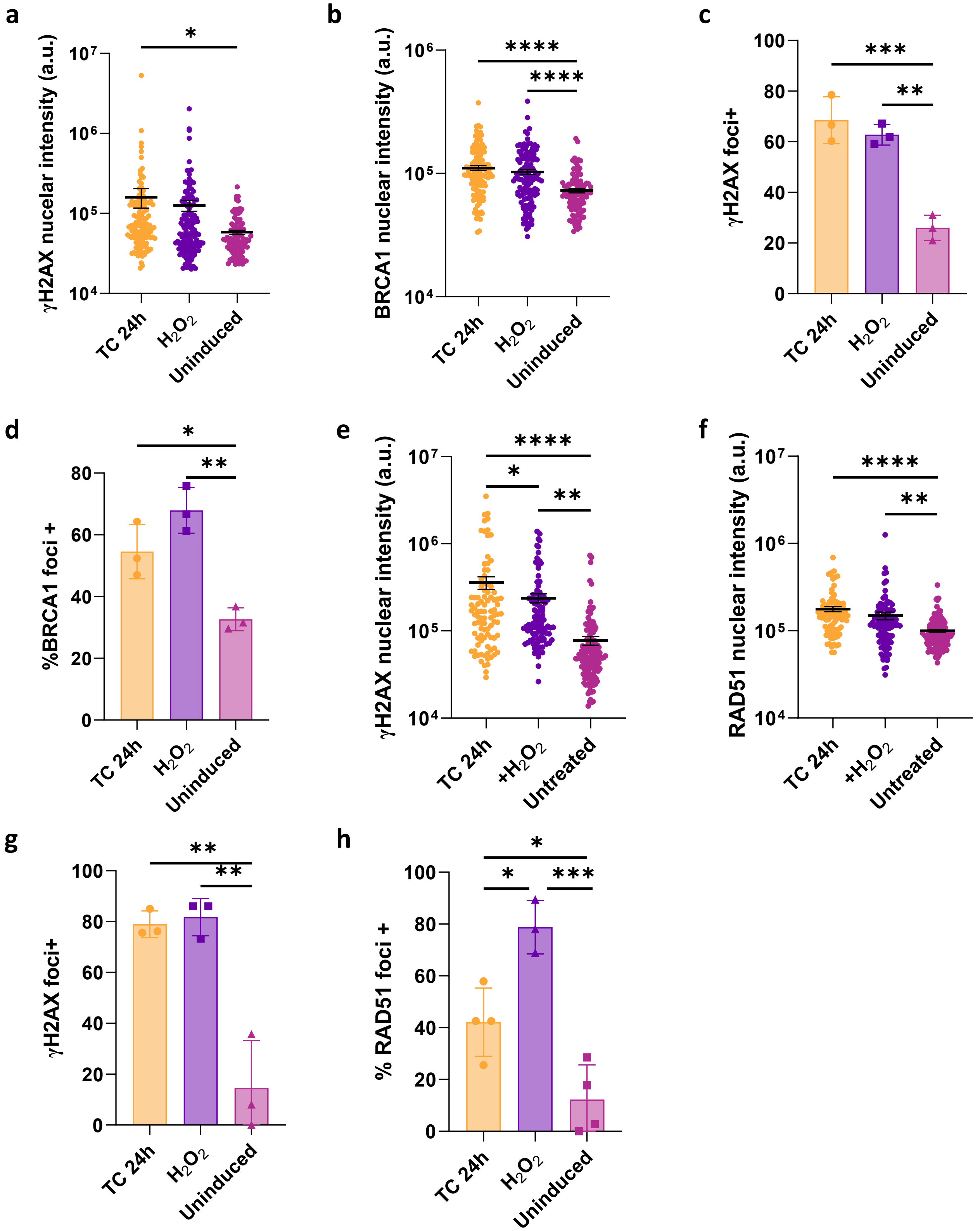

Supplementary Figure 8. Homologous recombination is impaired in TC expressing endothelial cells.

a Nuclear fluorescence intensity of γH2AX staining in cells treated with dox for 24h to induce TC expression or not, or treated with 30μM H_2O_2 as a positive control. These values correspond to the BRCA1 staining presented in **Fig 4a, b.**

b Nuclear fluorescence intensity of BRCA1 staining in cells treated with dox for 24h to induce TC expression or not, or treated with 30μM H_2O_2 as a positive control. These values correspond to the BRCA1 staining presented in **Fig 4a, b.**

c The frequency of endothelial cells positive for γH2AX foci staining in cells treated with dox for 24h to induce TC expression or not, or treated with 30μM H_2O_2 as a positive control. Cells with >10 foci were considered positive. These values correspond to the BRCA1 staining presented in **Fig 4a, c.**

d The frequency of endothelial cells positive for BRCA1 foci staining in cells treated with dox for 24h to induce TC expression or not, or treated with 30μM H_2O_2 as a positive control. Cells with >10 foci were considered positive. These values correspond to the BRCA1 staining presented in **Fig 4a, c.**

e Nuclear fluorescence intensity of γH2AX staining in cells treated with dox for 24h to induce TC expression or not, or treated with 30μM H_2O_2 as a positive control. These values correspond to the RAD51 staining presented in **Fig 4d, e.**

f Nuclear fluorescence intensity of RAD51 staining in cells treated with dox for 24h to induce TC expression or not, or treated with 30μM H_2O_2 as a positive control. These values correspond to the RAD51 staining presented in **Fig 4d, e.**

g The frequency of endothelial cells positive for γH2AX foci staining in cells treated with dox for 24h to induce TC expression or not, or treated with 30μM H_2O_2 as a positive control. Cells with >10 foci were considered positive. These values correspond to the RAD51 staining presented in **Fig 4d, f.**

h The frequency of endothelial cells positive for RAD51 foci staining in cells treated with dox for 24h to induce TC expression or not, or treated with 30μM H_2O_2 as a positive control. Cells with >10 foci were considered positive. These values correspond to the RAD51 staining presented in **Fig 4d, f.**

In all panels error bars show SEM, and significance was calculated by one-way ANOVA and Sidak's multiple comparisons test. *p<0.05, **p<0.01, ***p<0.001, ****p<0.0001.

Supplementary Figure 9

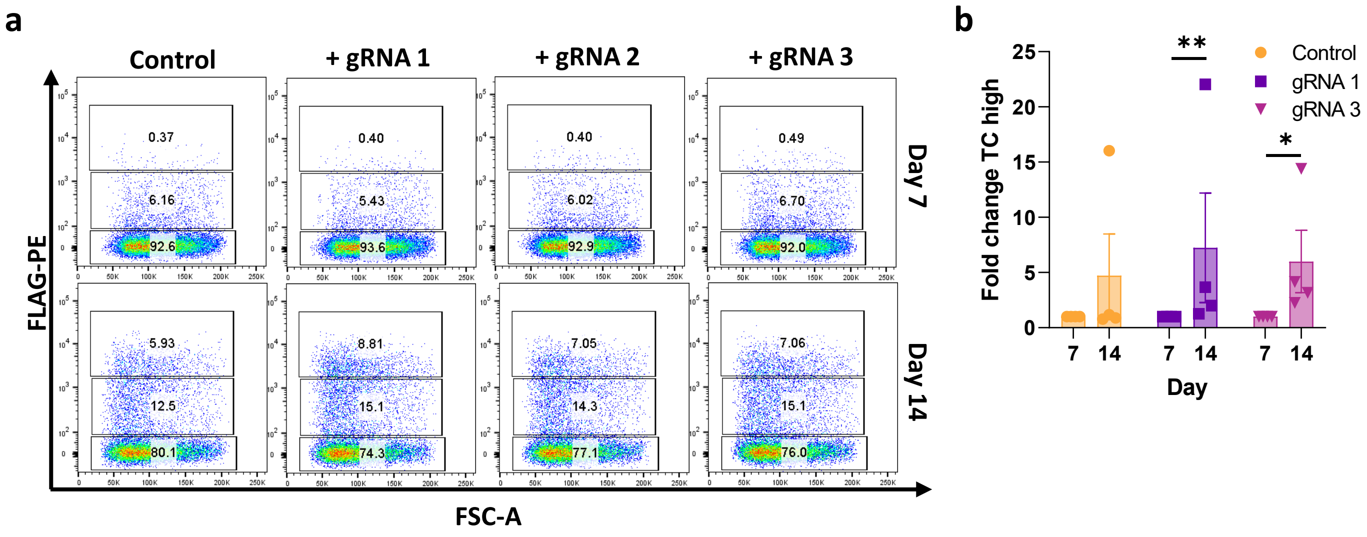

Supplementary Figure 9. *Cdkn2a* knockdown endothelial cells allows increased TC expression.

a Representative flow cytometry plots showing TC expression against FSC-A at day 14 after *Cdkn2a* knockout or not.

b Fold change of TC high population between days 7 and 14 after addition of dox in comparison to uninduced, *Cdkn2a* knockout endothelial cells, n=4. Statistical significance was calculated with a two-way ANOVA and Sidak's multiple comparisons test. All error bars show SEM, and *p<0.05, **p<0.01, ***p<0.001, ****p<0.0001.

Supplementary Figure 10

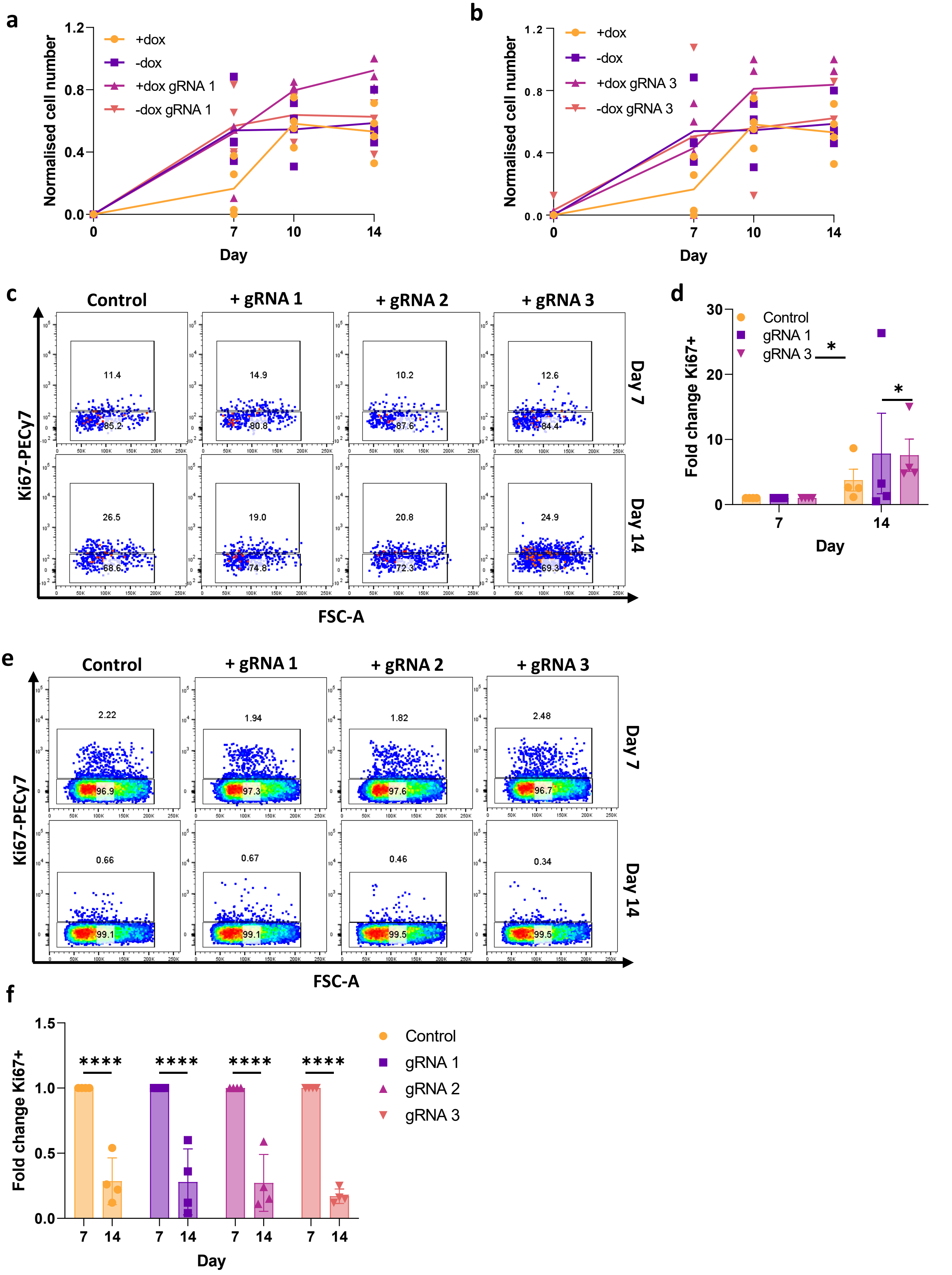

Supplementary Figure 10. *Cdkn2a* knockdown in TC expressing endothelial cells allows bypass of growth arrest.

a Normalised cell number in *Cdkn2a* knockout endothelial cells, treated or not with dox, over 14 days, using gRNA 1. Statistical significance was calculated with a two-way ANOVA and Sidak's multiple comparisons test, n=4.

b Normalised cell number in *Cdkn2a* knockout endothelial cells, treated or not with dox, over 14 days, using gRNA 3. Statistical significance was calculated with a two-way ANOVA and Sidak's multiple comparisons test, n=4. P-values for **a** and **b** are outlined in Supplementary Table 1.

c Representative flow cytometry plots showing Ki67 expression within the TC high population against FSC-A at day 7 and 14 after *Cdkn2a* knockout or not.

d Fold change of Ki67 expression within the TC high population between days 7 and 14 after addition of dox in comparison to uninduced, *Cdkn2a* knockout endothelial cells, n=4. Statistical significance was calculated with a two-way ANOVA and Sidak's multiple comparisons test.

e Representative flow cytometry plots showing Ki67 expression within the uninduced endothelial cell population against FSC-A at day 7 and 14 after *Cdkn2a* knockout or not.

f Fold change of Ki67 expression within the uninduced endothelial cell population between days 7 and 14 after addition of dox in comparison to uninduced endothelial cells, n=4. Statistical significance was calculated with a two-way ANOVA and Sidak's multiple comparisons test. In all panels error bars show SEM, and *$p<0.05$, **$p<0.01$, ***$p<0.001$, ****$p<0.0001$.

Supplementary Table 1

	Day 7	Day 10	Day 14
gRNA 1			
+dox v –dox	0.0010 **	0.9631	0.8904
+dox v +dox gRNA 1	0.0016 **	0.0769	0.0006 ***
+dox v –dox gRNA 1	0.0004 ***	0.8793	0.6269
gRNA 2			
+dox v –dox	<0.0001 ****	>0.9999	0.9992
+dox v +dox gRNA 2	<0.0001 ****	<0.0001 ****	<0.0001 ****
+dox v –dox gRNA 2	0.0004 ***	0.8008	0.8734
gRNA 3			
+dox v –dox	0.0025 **	0.9709	0.9118
+dox v +dox gRNA 3	0.0381 *	0.0816	0.0145 *
+dox v –dox gRNA 3	0.0060 **	0.9919	0.7128

P-values for normalised cell counts in *Cdkn2a* knockout experiments.

Supplementary Table 2: Antibodies used for immunofluorescence and flow cytometry experiments

Target	Species	Fluorochrome	Dilution	Application	Manufacturer
Primary					
FLAG	Rabbit/mouse	Unconjugated	1:500	IF	Sigma
γH2AX	Rabbit	Unconjugated	1:200	IF	Cell Signalling Technologies
RAD51	Mouse	Unconjugated	1:50	IF	Invitrogen
BRCA1	Rabbit	Unconjugated	1:750	IF	Invitrogen
p16	Rabbit	Unconjugated	1:100<br>1:500	IF<br>Flow cytometry	Abcam
R-loops	Mouse	Unconjugated	1:50	IF	Abcam
FLK1	Rat	PECy7	1:200<br>1:100	Flow cytometry<br>Cell sorting	BioLegend
TIE2	Rat	PE	1:200<br>1:100	Flow cytometry<br>Cell sorting	eBioscience
CD144	Rat	APC	1:100	Flow cytometry	eBioscience
CD31	Rat	PerCP-Cy5.5	1:100	Flow cytometry	BioLegend
FLAG	Rat	PE, BV421	1:800	Flow cytometry	BioLegend
γH2AX	Rabbit	APC	1:400	Flow cytometry	Cell Signalling Technologies
Ki67	Rat	PECy7	1:1000	Flow cytometry	eBioscience
Secondary					
Rabbit IgG	Goat	Alexa Fluor 647	1:800<br>1:2000	IF<br>Flow cytometry	Invitrogen
Rabbit IgG	Goat	Alexa Fluor Plus 555	1:800	IF	Invitrogen
Mouse IgG	Goat	Alexa Fluor 647	1:800	IF	Invitrogen
Mouse IgG	Goat	Alexa Fluor 555	1:800	IF	Invitrogen

Supplementary Table 3: Sequences of guide RNA used for CRISPR/Cas9 knockout of *Cdkn2a.*

gRNA #	Target	Sequence	Manufacturer
1	Cdkn2a	TGAGCTGAAGCTATGCCCGT	Integrated DNA Technologies
2	Cdkn2a	GGAAGGCTTCCTGGACACGC	Integrated DNA Technologies
3	Cdkn2a	GGGAACGTCGCCCAGACCGA	Integrated DNA Technologies
