# Supplementary material for: The oncogenic fusion protein TAZ::CAMTA1 promotes genomic instability and senescence through hypertranscription
Source: Commun Biol. 2023 Nov 18;6:1174. doi: 10.1038/s42003-023-05540-4 (PMC10657451; doi:10.1038/s42003-023-05540-4)
Supplement: Supplementary file 2 — Description of Additional Supplementary Files [file 42003_2023_5540_MOESM2_ESM.pdf]

Description of Additional Supplementary Files

File name: Supplementary Data
Description: All source data behind all graphs in the paper
