# Supplementary material for: The oncogenic fusion protein TAZ::CAMTA1 promotes genomic instability and senescence through hypertranscription
Source: Commun Biol. 2023 Nov 18;6:1174. doi: 10.1038/s42003-023-05540-4 (PMC10657451; doi:10.1038/s42003-023-05540-4)
Supplement: Supplementary file 4 — Reporting Summary [file 42003_2023_5540_MOESM4_ESM.pdf]

nature portfolio

Corresponding author(s): Valerie Kouskoff

Last updated by author(s): Oct 11, 2023

Reporting Summary

Nature Portfolio wishes to improve the reproducibility of the work that we publish. This form provides structure for consistency and transparency in reporting. For further information on Nature Portfolio policies, see our Editorial Policies and the Editorial Policy Checklist.

Statistics

For all statistical analyses, confirm that the following items are present in the figure legend, table legend, main text, or Methods section.

n/a	Confirmed	
☐	☒	The exact sample size (*n*) for each experimental group/condition, given as a discrete number and unit of measurement
☐	☒	A statement on whether measurements were taken from distinct samples or whether the same sample was measured repeatedly
☐	☐	The statistical test(s) used AND whether they are one- or two-sided<br>*Only common tests should be described solely by name; describe more complex techniques in the Methods section.*
☒	☐	A description of all covariates tested
☐	☒	A description of any assumptions or corrections, such as tests of normality and adjustment for multiple comparisons
☐	☒	A full description of the statistical parameters including central tendency (e.g. means) or other basic estimates (e.g. regression coefficient) AND variation (e.g. standard deviation) or associated estimates of uncertainty (e.g. confidence intervals)
☐	☒	For null hypothesis testing, the test statistic (e.g. *F*, *t*, *r*) with confidence intervals, effect sizes, degrees of freedom and *P* value noted<br>*Give P values as exact values whenever suitable.*
☒	☐	For Bayesian analysis, information on the choice of priors and Markov chain Monte Carlo settings
☒	☐	For hierarchical and complex designs, identification of the appropriate level for tests and full reporting of outcomes
☒	☐	Estimates of effect sizes (e.g. Cohen's *d*, Pearson's *r*), indicating how they were calculated

Our web collection on statistics for biologists contains articles on many of the points above.

Software and code

Policy information about availability of computer code

Data collection	Micro-Manager v2.0 (image acquisition)<br>Leica LAS X (image acquisition)<br>BD FACSDiva v9.0 (flow cytometry data acquisition)
Data analysis	GraphPad Prism v9<br>FlowJo v10<br>ImageJ v1.53o<br>Gene Set Enrichment Analysis v4.2.3<br>Ingenuity Pathway Analysis

For manuscripts utilizing custom algorithms or software that are central to the research but not yet described in published literature, software must be made available to editors and reviewers. We strongly encourage code deposition in a community repository (e.g. GitHub). See the Nature Portfolio guidelines for submitting code & software for further information.

Data

Policy information about availability of data

All manuscripts must include a data availability statement. This statement should provide the following information, where applicable:

- Accession codes, unique identifiers, or web links for publicly available datasets
- A description of any restrictions on data availability
- For clinical datasets or third party data, please ensure that the statement adheres to our policy

Provide your data availability statement here.

Human research participants

Policy information about studies involving human research participants and Sex and Gender in Research.

Reporting on sex and gender	*Use the terms sex (biological attribute) and gender (shaped by social and cultural circumstances) carefully in order to avoid confusing both terms. Indicate if findings apply to only one sex or gender; describe whether sex and gender were considered in study design whether sex and/or gender was determined based on self-reporting or assigned and methods used. Provide in the source data disaggregated sex and gender data where this information has been collected, and consent has been obtained for sharing of individual-level data; provide overall numbers in this Reporting Summary. Please state if this information has not been collected. Report sex- and gender-based analyses where performed, justify reasons for lack of sex- and gender-based analysis.*
Population characteristics	*Describe the covariate-relevant population characteristics of the human research participants (e.g. age, genotypic information, past and current diagnosis and treatment categories). If you filled out the behavioural & social sciences study design questions and have nothing to add here, write "See above."*
Recruitment	*Describe how participants were recruited. Outline any potential self-selection bias or other biases that may be present and how these are likely to impact results.*
Ethics oversight	*Identify the organization(s) that approved the study protocol.*

Note that full information on the approval of the study protocol must also be provided in the manuscript.

Field-specific reporting

Please select the one below that is the best fit for your research. If you are not sure, read the appropriate sections before making your selection.

☒ Life sciences ☐ Behavioural & social sciences ☐ Ecological, evolutionary & environmental sciences

For a reference copy of the document with all sections, see nature.com/documents/nr-reporting-summary-flat.pdf

Life sciences study design

All studies must disclose on these points even when the disclosure is negative.

Sample size	All experiments were subject to at least 3 independent/biological repeats, as indicated for each panel in the figure legend. No sample size calculation was performed.
Data exclusions	For RNA-sequencing experiments, a cut off of $p<0.01$ and a log2 fold change of <-1 or >1 was used to define differentially expressed genes for GSEA and IPA analysis.
Replication	All experiments were independently replicated multiple times, as indicated in the figure legends. All attempts at replication were successful.
Randomization	Samples were not randomised during this study.
Blinding	Investigators were not blinded during data collection or analysis.

Reporting for specific materials, systems and methods

We require information from authors about some types of materials, experimental systems and methods used in many studies. Here, indicate whether each material, system or method listed is relevant to your study. If you are not sure if a list item applies to your research, read the appropriate section before selecting a response.

Materials & experimental systems

n/a	Involved in the study
☐	☒ Antibodies
☐	☒ Eukaryotic cell lines
☒	☐ Palaeontology and archaeology
☒	☐ Animals and other organisms
☒	☐ Clinical data
☒	☐ Dual use research of concern

Methods

n/a	Involved in the study
☒	☐ ChIP-seq
☐	☒ Flow cytometry
☒	☐ MRI-based neuroimaging

Antibodies

Antibodies used

Primary antibodies for immunofluorescence
FLAG (mouse), M2 (Sigma, #F1804)
FLAG (rabbit), polyclonal (Sigma, #F7425)
yH2AX, 20E3 (Cell Signalling Technology, #9718)
RAD51, 3C10 (Invitrogen, #11365053)
BRCA1, 6B4 (Invitrogen, #11561450)
p16, EPR20418 (Abcam, ab211542)
R-loops, S9.6 (Merck, #MABE1095)

Secondary antibodies for immunofluorescence
Anti-rabbit IgG-AF647 (Invitrogen #A21244)
Anti-rabbit IgG-AF555 Plus (Invitrogen #A32732)
Anti-mouse IgG-AF647 (Invitrogen #A32728)
Anti-mouse IgG-AF555 (Invitrogen #A21422)

Conjugated primary antibodies for flow cytometry/cell sorting
FLK1-PECy7, Avas12 (eBioscience, #136414)
TIE2-PE, TEK4 (eBioscience #12-5987-82)
CD144-APC, BV13 (eBioscience #17-1441-82)
CD31-PerCP-Cy5.5, 390 (BioLegend, #102419)
Ki67-PECy7, SolA15 (eBioscience #25-5698-82)
FLAG-PE, L5 (BioLegend #637309)

The species in which these antibodies were raised and the concentrations used are stated in the methods section.

Validation

All antibodies used were purchased from commercial suppliers (as indicated) and were validated for the relevant applications and target species in this study by the respective manufacturers (flow cytometry or immunofluorescence).

Eukaryotic cell lines

Policy information about cell lines and Sex and Gender in Research

Cell line source(s)	Ainv18 mouse embryonic stem cells
Authentication	*Describe the authentication procedures for each cell line used OR declare that none of the cell lines used were authenticated.*
Mycoplasma contamination	Cell line used tested negative for mycoplasma
Commonly misidentified lines (See ICLAC register)	Cell line used is not commonly misidentified

Flow Cytometry

Plots

Confirm that:

☒ The axis labels state the marker and fluorochrome used (e.g. CD4-FITC).

☒ The axis scales are clearly visible. Include numbers along axes only for bottom left plot of group (a 'group' is an analysis of identical markers).

☒ All plots are contour plots with outliers or pseudocolor plots.

☒ A numerical value for number of cells or percentage (with statistics) is provided.

Methodology

Sample preparation	Ainv18 mESCs differentiated into endothelial cells. No tissue processing was carried out.
Instrument	BD LSRFortessa
Software	BD FACSDiva v9.0
Cell population abundance	*Describe the abundance of the relevant cell populations within post-sort fractions, providing details on the purity of the samples and how it was determined.*
Gating strategy	FSC-A v SSC-A plots were used to gate cells, then FSC-W v FSC-A plots to gate out doublets. Gating for positive/negative staining were based on unstained control samples.

☐ Tick this box to confirm that a figure exemplifying the gating strategy is provided in the Supplementary Information.
